# MCT-1/miR-34a/IL-6/IL-6R signaling axis promotes EMT progression, cancer stemness and M2 macrophage polarization in triple-negative breast cancer

**DOI:** 10.1186/s12943-019-0988-0

**Published:** 2019-03-18

**Authors:** Yueh-Shan Weng, Hong-Yu Tseng, Yen-An Chen, Pei-Chun Shen, Aushia Tanzih Al Haq, Li-Mei Chen, Yi-Chung Tung, Hsin-Ling Hsu

**Affiliations:** 1 Institute of Molecular and Genomic Medicine, National Health Research Institutes, 35 Keyan Road, Zhunan, Miaoli County, 35053 Taiwan; 2 Research Center for Applied Sciences, Academia Sinica, Taipei, Taiwan

**Keywords:** MCT-1/miR-34a/IL-6/IL-6R pathway, EMT, M2 macrophages, Cancer stemness

## Abstract

**Background:**

Triple-negative breast cancer (TNBC) is a poor prognostic breast cancer with the highest mutations and limited therapeutic choices. Cytokine networking between cancer cells and the tumor microenvironment (TME) maintains the self-renewing subpopulation of breast cancer stem cells (BCSCs) that mediate tumor heterogeneity, resistance and recurrence. Immunotherapy of those factors combined with targeted therapy or chemoagents may advantage TNBC treatment.

**Results:**

We found that the oncogene **M**ultiple **C**opies in **T**-cell Malignancy **1** (MCT-1/MCTS1) expression is a new poor-prognosis marker in patients with aggressive breast cancers. Overexpressing MCT-1 perturbed the oncogenic breast epithelial acini morphogenesis and stimulated epithelial-mesenchymal transition and matrix metalloproteinase activation in invasive TNBC cells, which were repressed after MCT-1 gene silencing. As mammary tumor progression was promoted by oncogenic MCT-1 activation, tumor-promoting M2 macrophages were enriched in TME, whereas M2 macrophages were decreased and tumor-suppressive M1 macrophages were increased as the tumor was repressed via MCT-1 knockdown. MCT-1 stimulated interleukin-6 (IL-6) secretion that promoted monocytic THP-1 polarization into M2-like macrophages to increase TNBC cell invasiveness. In addition, MCT-1 elevated the soluble IL-6 receptor levels, and thus, IL-6R antibodies antagonized the effect of MCT-1 on promoting M2-like polarization and cancer cell invasion. Notably, MCT-1 increased the features of BCSCs, which were further advanced by IL-6 but prevented by tocilizumab, a humanized IL-6R antibody, thus MCT-1 knockdown and tocilizumab synergistically inhibited TNBC stemness. Tumor suppressor miR-34a was induced upon MCT-1 knockdown that inhibited IL-6R expression and activated M1 polarization.

**Conclusions:**

The MCT-1 pathway is a novel and promising therapeutic target for TNBC.

**Electronic supplementary material:**

The online version of this article (10.1186/s12943-019-0988-0) contains supplementary material, which is available to authorized users.

## Background

Cancer cell-intrinsic mechanisms and cell-extrinsic factors determine tumor development and aggressiveness [1]. Epithelial-mesenchymal transition (EMT) induces epithelial cells transforming into mesenchymal cells [2], cancer cell movement, cancer progression, metastasis and stemness. Tumor-associated macrophages (TAMs) derived from peripheral blood monocytes are recruited to microenvironment and polarized into M1 or M2 macrophages in response to secreted factors from cancer cells or microenvironmental cells [3]. M1 macrophages highly express inducible nitric oxide synthase (iNOS) and Tumor Necrosis Factor (TNF)-α and promote pro-inflammatory and immune responses that prevent oncogenic effects [4], while M2 macrophages express Arginase 1 (ARG1) and highly produce cytokines, growth factors and protease that are crucial for pro-tumorigenic processes. Furthermore, M2 macrophages stimulate tumor angiogenesis [5, 6], cancer cell migration/invasion, immunosuppression and marix remodeling.

IL-6 is involved in immune regulation [7], inflammation and oncogenesis. IL-6 and IL-6R interaction induces the dimerization of glycoprotein 130 (gp130) to activate signal transducer and activator of transcription 3 (Stat3) [8]. However, signaling activation of IL-6/Stat3 repressed the p53-mediated miR-34a expression [8], and miR-34a-deficient mice exhibited the stimulation of Stat3, IL-6R and Snail as well as the increase in colorectal cancer invasion/metastasis. IL-6 induction is associated with a poorer prognosis in patients with breast cancer and serum IL-6 levels are increased with pathological grades [9]. Clinical trials of IL-6 and IL-6R immunotherapy have been widely studied in multicentric Castleman’s disease [7], multiple myeloma and solid tumors including renal prostate, lung, colorectal and ovarian cancers. However, the studies of IL-6/IL-6R/gp130 immunotherapy for treatment of breast cancers are limited. IL-6/IL-6R/gp130 pathway communicates between breast tumor and immune cells [10], resulting in tumor promotion and enriched effect on BCSCs. Therefore, targeting IL-6/Sta3 signaling axis potentially improve the efficacy of cancer immunotherapy [11]. Supporting this notion, the nanoparticles of anti- CD44 Ab encapsulating anti-IL-6R Ab target the TME and CD44^+^ BCSCs that inhibit the metastatic niche of triple-negative and luminal breast cancer in two mouse models, namely syngeneic BALB/c mice bearing 4 T1 cells and transgenic MMTV-PyMT mice [12]. Also, the use of nanoparticle-based system for CD44 and IL-6R immunotherapy suppresses Stat3, Sox2, VEGF-A, MMP-9 and CD206 expression in breast tissues as well as Sox2^+^/CD206^+^ stem cells in lung metastatic foci.

MCT-1 is a ribosome binding protein encoded by MCTS1 gene [13, 14], which orchestrates ribosomal recycling, translation reinitiation and tissue growth. Density regulated protein and the MCT-1 heterodimer bind to the 40S ribosomal subunit and, with the recruitment of tRNA, cooperatively regulate noncanonical translation initiation [15, 16]. Moreover, MCT-1 affects mitotic progression via interacting with γ-tubulin molecule and Src/p190B complex [17, 18]. MCT-1 destabilizes p53 and PTEN in a ubiquitin-dependent proteasome pathway [18, 19]. Consequently, MCT-1 expression advances the p53-null or PTEN-null cancer cell progression and chromosomal/nuclear aberrations [17, 18, 20, 21]. Importantly, targeting MCT-1 suppresses genomic instability and tumorigenicity [18, 22]. MCT-1 overexpression also induces ROS generation [23], leading to YY-1/EGFR/MnSOD signaling amplification and cancer cell invasion. Here, we first demonstrate that MCT-1 induces IL-6/IL-6R/Stat3 pathway, M2 macrophage polarization, TNBC progression and stemness.

## Results

### MCT-1 is a poor-prognosis marker of aggressive breast cancer

Oncogenic MCT-1 (also known as MCTS1) activation in breast cancer was investigated using the Kaplan-Meier Plotter database [24], and we observed that high MCT-1 expression in patients was associated with lower overall survival (OS) in overall breast cancer (*p* = 0.0053) as well as in TP53 wild type (*p* = 0.024) (Additional file 1: Figure S1A), lymph node metastasis-free (*p* = 0.001), HER2-negative (*p* = 0.0067), luminal-A (*p* = 0.026) and luminal-B (*p* = 0.043) breast cancers than that of patients with low-level MCT-1. Patients with MCT-1 gene overexpression also exhibited lower recurrence-free survival (RFS) in overall breast cancer (*p* = 1E-16) as well as in TP53 wild type (*p* = 0.029) (Additional file 1: Figure S1B), lymph node metastasis (*p* = 0.017) and metastasis-free (*p* = 0.00096), ER-negative (*p* = 0.015), HER2-negative (*p* = 1E-06), TNBC (*p* = 0.046), luminal-A (*p* = 5E-08) and luminal-B (*p* = 2.3E-06) breast cancer than did patients with low MCT-1 levels.

**Fig. 1:**
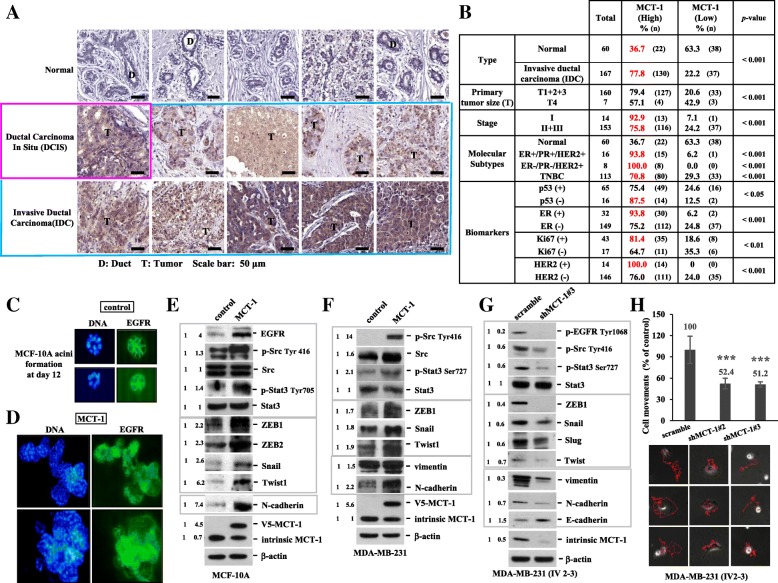
MCT-1 is a prognostic marker of human breast carcinoma**.** (**a**) Immunohistology was used to analyze MCT-1 protein levels in normal tissues, DCIS and IDC of the breast. T: tumor. D: Duct. Scale bars, 50 μm. (**b**) MCT-1 protein levels in breast cancers were classified based on pathologic stages, molecular subtypes and biomarkers. The results were evaluated from 6 to 8 randomly chosen imaging fields per sample. The Fisher exact probability test of independence was used to calculate the clinical significance. (**c** and **d**) MCF-10A acini morphogenesis was studied on a Matrigel matrix culture for 12 days followed by EGFR Ab (green) and DNA (blue) staining. (**e** and **f**) EGFR expression, Src/Stat3 activation and EMT molecules were analyzed in MCF-10A and MDA-MB-231 cells without (control) or with MCT-1 induction (MCT-1). (**g**) EMT molecules and EGFR/Src/Stat3 activation were assessed in MDA-MB-231 (IV2–3) without (scramble) or with MCT-1 knockdown (shMCT-1#3). (**h**) Time-lapse imaging recorded movements of MDA-MB-231 (IV2–3) (scramble vs. shMCT-1#2 and #3). Different blots were normalized with β-actin and then the relative protein amounts were compared with the corresponding controls. One-way ANOVA with a post hoc two-tailed t-test was used to calculate the statistical significance of the cell migration assays (*n* = 12). (*** *p* < 0.001)

Immunohistostaining revealed MCT-1 protein enrichment in ductal carcinoma in situ (DCIS) and in invasive ductal carcinoma (IDC) of the breast comparative to that in normal breast tissue and the adjacent stroma (Fig. 1a). Moreover, MCT-1 protein was often enriched in IDC (77.8%, *n* = 167) as well as in the majority of patients at stage I (92.9%, *n* = 14) and stage II/III (75.8%, *n* = 153) but was less identified in normal breast tissues (36.7%, *n* = 60) (Fig. 1b). Characterized by the molecular subtypes, we found MCT-1 induction in 93.8% of ER^+^/PR^+^/HER2^+^ cancers (n = 16), 100% of ER^−^/PR^−^/HER2^+^ (*n* = 8) cancers and 70.8% of TNBC (ER^−^/PR^−^/HER2^−^) (*n* = 113). MCT-1 protein is also enriched in p53-negative (87.5%, n = 16), ER-positive (93.8%, *n* = 32), Ki67-positive (81.4%, *n* = 43) and HER2-positive (100%, *n* = 14) breast carcinomas. Conceivably, MCT-1 gene activation and protein increase implicate in breast carcinogenesis.

### MCT-1 promotes mammary acini oncogenesis, EMT progression and MMP activation

Normal breast epithelial MCF-10A cells grown on a basement membrane matrix form polarized, growth-arrested acini-like spheroids that recapitulate the glandular architecture in vivo [25]. Examining MCF-10A acini morphogenesis on a growth factor-reduced Matrigel for 12 days, the control cells assembled in a regular acini-like, spheroid with well-organized nuclear arrangement and cell-cell interaction (Fig. 1c). MCF-10A cells were virally transfected with pLXSN or pLXSN/V5-MCT-1 (V5-tagged MCT-1) using the retroviral supernatants. Upon MCT-1 overexpression (V5-MCT-1), MCF-10A acinarization was perturbed with aggressive cellular growth and proliferation that produced a multiacinar structure with a disorganized nuclear arrangement and poor cell-cell interaction (Fig. 1d), implying that MCT-1 overexpression disturbed the early stage of mammary acinar morphogenesis. Furthermore, the development of MCF-10A acini on Matrigel matrix at day 3 and day 5 were examined (Additional file 2: Figure S2A and B). The diameters of MCF-10A acini at day 5 were quantified (Additional file 2: Figure S2C), and it showed that MCT-1 overexpression more promoted MCF-10A acini development than control cells.

EMT disrupts MCF-10A polarity and growth-arrested spheroid formation [26]. Consistently, overexpressing MCT-1 in MCF-10A cells induced EGFR (Fig. 1e), phospho-activation of Src (p-Src Tyr416) and Stat3 (p-Stat3 Tyr705), EMT transcription factors (ZEB1, ZEB2, Snail, Twist1) and N-cadherin. Moreover, MCT-1 overexpression in human TNBC (MDA-MB-231) cells increased Src/Stat3 activation as well as the levels of ZEB1 (Fig. 1f), Snail, Twist1, vimentin and N-cadherin. Conversely, MCT-1 gene was targeted by a small hairpin RNA (shMCT-1) in a highly invasive MDA-MB-231 (IV2–3) subline that has been isolated from two rounds of in vivo selection from lung metastases of the parental MDA-MB-231 cells [27]. Silencing MCT-1 (shMCT-1#3) repressed EGFR/Src activation as well as the levels of ZEB1 (Fig. 1g), Snail, Slug, Twist, vimentin and N-cadherin but increased E-cadherin. Similar results were also identified in the shMCT-1#2 clone of IV2–3 (Additional file 2: Figure S2D). Corresponding to mesenchymal-epithelial transition, the migratory abilities were significantly reduced in the shMCT-1 clones (#2 and #3) compared with those scramble control cells (Fig. 1h).

An examination of precursor (pro) and active matrix metalloproteinases (MMPs) and their inhibitors (TIMPs) revealed that MCT-1 increased the amounts of vascular endothelial growth factor (VEGF) alongside pro- and active- MMP2 and MMP9 elevation but reduced TIMP-1 and TIMP-2 levels (Additional file 3: Figure S3A). However, shMCT-1#3 increased TIMP-1 and TIMP-2 but decreased pro- and active- MMP2 and MMP9 (Additional file 3: Figure S3B). Consistently, MCT-1 promoted MDA-MB-231 invasiveness through a Boyden chamber-coating Matrigel matrix (Additional file 3: Figure S3C), whereas shMCT-1#3 repressed the high invasive ability of MDA-MB-231 (IV2–3) (Additional file 3: Figure S3D). Using gelatin zymography to evaluate gelatinases in the conditioned medium (CM) (Additional file 3: Figure S3E), induced MMP2 and MMP9 activities were detected while MCT-1 overexpressing but these activities were decreased in shMCT-1#3 condition. Likewise, shMCT-1 (#3–28) inhibited the wound closure ability (Additional file 3: Figure S3G) and the invasiveness of murine TNBC (4 T1) cells (Additional file 3: Figure S3H) alongside reducing MMP2 and MMP9 but increasing TIMP-1 and TIMP-3 (Additional file 3: Figure S3F). Confirmatively, MCT-1 promotes EMT processes and MMP activities which enhance cancer cell migration/invasion.

### MCT-1 induces the IL-6/IL-6R signaling

**Fig. 2:**
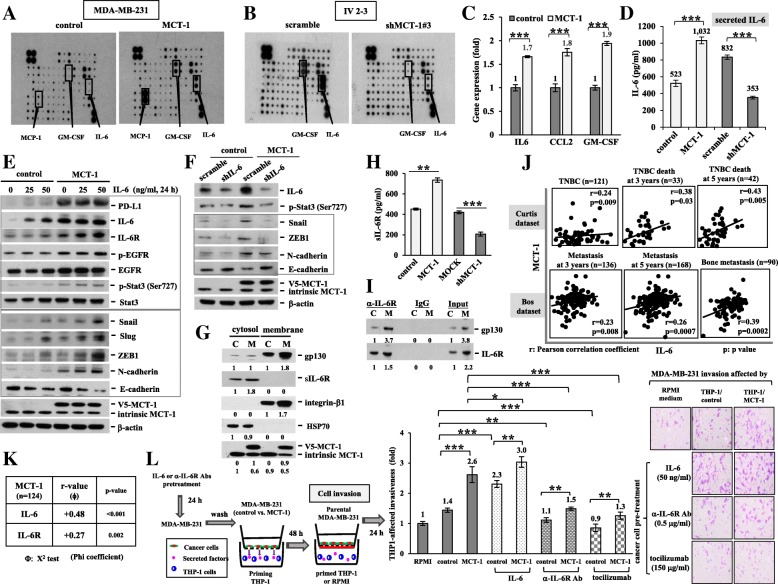
The MCT-1/IL-6/IL-6R pathway activates THP-1 differentiation that stimulates cancer cell invasion. (**a** and **b**) The cytokine arrays were incubated with the CM of the MDA-MB-231 cells (control vs. MCT-1) and IV2–3 subline (scramble vs. shMCT-1#3) to identify the secreted factors specific to the indicated cellular conditions. (**c**) Quantitative RT-PCR evaluated IL-6, CCL2 and GM-CSF mRNA levels in the MDA-MB-231 cells. (**d**) The IL-6 secreted from the MDA-MB-231 cells (control vs. MCT-1) and IV2–3 subline (scramble vs. shMCT-1#3) were quantified by ELISA using the anti-IL-6 mAb. The results were normalized with the plated cell numbers (2 × 10^5^). (**e**) PD-L1, IL-6/IL-6R, EGFR, Stat3 and EMT molecules were analyzed upon different doses of IL-6 simulation for 24 h. Different blots with normalized internal control were used to present the data of the experiment. (**f**) The effect of IL-6 knockdown (shIL-6) on EMT signaling molecules were characterized. (**g**) The proteins in the cytosol and membrane fractions were isolated from 5 × 10^6^ cells and then compared between control (**c**) and MCT-1 (**m**) groups. (**h**) The conditioned media of the MDA-MB-231 cells with different MCT-1 expression conditions were harvested after 48 h culture. The ELISA measured soluble IL-6R (sIL-6R) levels, and the results were normalized with the plated cell numbers. (**i**) The interaction of gp130 and IL-6R in vivo were identified by IL-6R immunoprecipitation assay. (**j**) The clinical relevance of MCT-1 and IL-6 was examined in TNBC (Curtis database) and in metastatic breast cancer (Bos dataset) using the Oncomine database. Pearson’s correlation coefficient indicates the statistical significance. (**k**) MCT-1 associated with IL-6/IL-6R was studied using the breast cancer cDNA arrays (*n* = 124). The Chi-square test was used to calculate the statistical significance. (**l**) The MDA-MB-231 cells were pretreated by IL-6, anti-IL-6R mAb or tocilizumab for 24 h followed by THP-1 coculture in the transwell for 48 h. Parental MDA-MB-231 invasiveness was examined as cocultured with the preconditioned THP-1 cells in a Boyden chamber-coating Matrigel matrix for 24 h. The results are expressed as the mean ± SD of three independent assays (*n* = 3). One-way ANOVA with a post hoc two-tailed t-test was used to calculate the statistical significance of pairwise comparisons. (*p < 0.05; **p < 0.01; *** *p* < 0.001)

The cytokine arrays were incubated with the CM of the MDA-MB-231 cells to identify the factors that communicate between cancer cells and immune cells. The results exhibited that there were more IL-6, CCL2 and GM-CSF secretion from MCT-1-overexpressing cells relative to control cells (Fig. 2a). However, only IL-6 and GM-CSF secretion were dramatically reduced after loss-function of MCT-1 (shMCT-1#3) compared with that of scramble control of the high-invasive IV2–3 cells (Fig. 2b). The IL-6, CCL2 and GM-CSF mRNA levels induced by MCT-1 were also detected by quantitative RT-PCR (qRT-PCR) (Fig. 2c). An enzyme-linked immunosorbent assay (ELISA) with the specific anti-IL-6 Ab confirmed that IL-6 secretion was promoted by MCT-1 overexpression (Fig. 2d), but it was significantly repressed in shMCT#3 cells.

The IL-6-STAT3-Twist circuit stimulates the EMT process and M2 macrophage polarization [28]. We found that MCT-1 increased the programmed death-ligand 1 (PD-L1), IL-6 and IL-6R amounts (Fig. 2e), and IL-6 further advanced MCT-1-induced EGFR and Stat3 phospho-activation as well as Snail, Slug, ZEB1 and N-cadherin but further suppressed E-cadherin in a dose-dependent manner. MCT-1 and IL-6 additively stimulated EMT molecules (EGFR, p-Stat3, Snail, Slug, ZEB1 and N-caherin), and PD-L1 was induced by MCT-1 but not further stimulated by IL-6. Silencing IL-6 (shIL-6) expression also reduced the Stat3 activation and EMT signaling molecules (Snail, ZEB1 and N-cadherin) but induced E-cadherin in the MCT-1-overexpressing cells (Fig. 2f). Thus, MCT-1 enhances EMT process via IL-6 pathway. Furthermore, subcellular fractionation identified the levels of membrane-bound gp130 and soluble IL-6R (sIL-6R) were most elevated (Fig. 2g) and sIL-6R amounts present in the CM were also induced (Fig. 2h) upon MCT-1 overexpression. We speculate that MCT-1 regulates the proteolytic cleavage of IL-6R or the alternative splicing of IL-6R mRNA [29], by which amplifies IL-6 trans-signaling through sIL-6R rather than classic IL-6 signaling by membrane-bound IL-6R [30]. In the IL-6R pull-down assay, gp130 was increased by 3.7 fold while IL-6R was increased by 1.5 fold in the MCT-1-overexpressing cells (M) over the control cells (C) (Fig. 2i), indicating that MCT-1 overexpression induced more gp130 molecules interaction with IL-6R in vivo.

An assessment of the clinical connection between MCT-1 and IL-6, the positive correlations between MCT-1 and IL-6 gene activation in overall TNBC patients (r = 0.24, *p* = 0.009) as well as in TNBC patient deaths at 3 years (r = 0.38, *p* = 0.03) and at 5 years (r = 0.43, *p* = 0.005) were recognized in the Curtis dataset of the Oncomine database (Fig. 2j). Moreover, MCT-1 and IL-6 gene promotion was correlated in breast cancer patients with 3-year metastasis (r = 0.23, *p* = 0.008), 5-year metastasis (r = 0.26, *p* = 0.007) and bone metastasis (r = 0.39, *p* = 0.0002, *n* = 90) in the Bos dataset. Further analysis of the breast cancer cDNA arrays (*n* = 124) confirmed positive correlations of MCT-1 expression with that of IL-6 (r = 0.48, *p* < 0.001) and IL-6R (r = 0.27, *p* = 0.002) (Fig. 2k).

To inspect whether cancer cell invasion is affected by the macrophages, the MDA-MB-231 cells were pretreated with IL-6 (Fig. 2l), the anti-IL-6R mAb or tocilizumab before priming THP-1 monocytes. The preconditioned THP-1 cells were placed in the lower chamber to test the invasion ability of MDA-MB-231 cells from the upper chamber. The cell invasiveness was more enhanced by the THP-1 which were primed by MCT-1-overexpressing cells (THP-1/MCT-1) than which were primed by the control cells (THP-1/control). Furthermore, the invasiveness was advanced with THP-1 priming by IL-6-stimulated MDA-MB-231, whereas the pretreatment with the anti-IL-6R mAb or with tocilizumab failed to activate THP-1, thus suppressing the invasion.

### MCT-1 promotes mammary tumor progression and TAM polarization

**Fig. 3:**
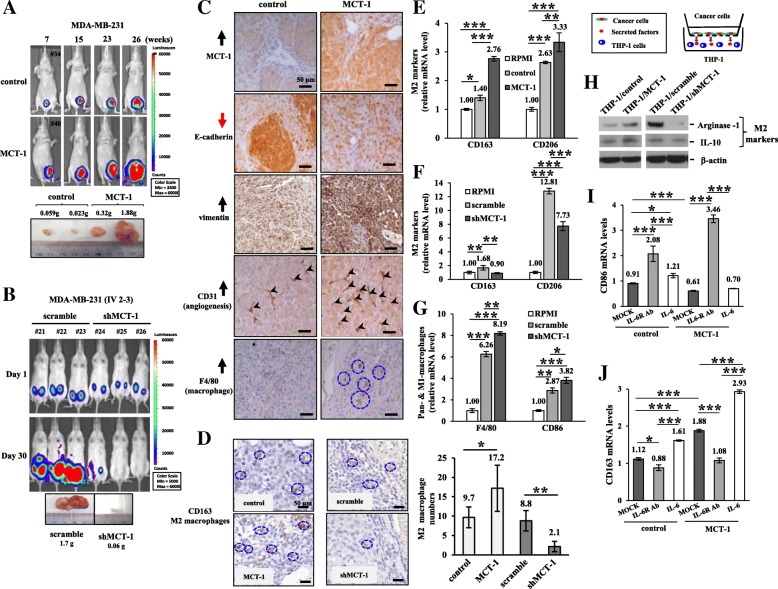
MCT-1 promotes breast tumor progression and M2 macrophage polarization**.** (**a** and **b**) MDA-MB-231 (control vs. MCT-1) and IV2–3 (scramble vs. shMCT-1#3) cells were injected into the mammary fat pad of nude mice. Tumor growth and burdens were analyzed at the indicated time. (**c**) Tumor immunohistology revealed the levels of MCT-1, E-cadherin, vimentin, CD31 (indicated by arrowheads) and F4/80 (enclosed in circles). Scale bars, 50 μm. (**d**) Immunohistochemistry characterized the tumor-associated CD163-positive M2 macrophages (enclosed in circles). The images were captured with 40X objective lens and macrophage numbers were counted (*n* = 6). (E and F) Levels of the M2-specific markers CD163 and CD206 were compared after THP-1 coculture with RPMI medium, MDA-MB-231 (control vs. MCT-1) or IV2–3 (scramble vs. shMCT-1#3) cells for 48 h. (**g**) The pan-macrophage (F4/80) and M1-like macrophage (CD86) markers were analyzed after THP-1 coculture with RPMI or with MDA-MB-231 cells (scramble vs. shMCT-1#3) for 48 h in a Boyden chamber. (**h**) THP-1 polarization into M2-like macrophages was signified by an increase in Arginase-1 and IL-10 after coculture with the indicated cells (control vs. MCT-1; scramble vs. shMCT-1#3). Different blots with normalized internal control were used to present the data of the experiment. (I and J) Quantitative RT-PCR analysis evaluated the CD86 and CD163 expression after THP-1 priming by RPMI or by the MDA-MB-231 cells (control vs. MCT-1) pretreated with an IL-6R mAb (tocilizumab) or stimulated with IL-6 for 48 h. The results are expressed as the mean ± SD (n = 3). One-way ANOVA with a post hoc two-tailed t-test was used to calculate the statistical significance of pairwise comparisons. (*p < 0.05; **p < 0.01; *** *p* < 0.001)

The bioluminescent MDA-MB-231 cells (1 × 10^5^) bearing a pGL3-luciferase reporter were injected into the mammary fat pad of immunodeficient BALB/c nude mice and monitored by an in vivo imaging system (Fig. 3a). At week 26, the MCT-1-overexpressing tumor burdens were dramatically increased up to 26-fold in the maximal tumor masses compared with those of the controls. However, MCT-1 knockdown (shMCT-1#3) greatly reduced the tumor development of aggressive IV2–3 cells (1 × 10^6^) after inoculation for 30 days (Fig. 3b), and the greatest difference in shMCT-1 tumor burdens showed a 28.3-fold reduction relative to scramble groups. MCT-1 and vimentin were increased but E-cadherin was reduced (Fig. 3c), together with enriched angiogenesis (CD31) and macrophages (F4/80) in the TME, as detected by immunohistostaining.

Tumor progression is prompted by M2 macrophages but repressed by M1 macrophages [31]. When the M1 marker (CD80) was assessed by immunohistochemistry, the CD80-positive M1 macrophages were more accumulated in the stroma of shMCT-1#3 tumors than in the scramble shRNA treated tumors (Additional file 4: Figure S4A). However, the CD80-positive M1 macrophages present in the stroma of MCT-1-overexpressing tumors were less than the control group (Additional file 4: Figure S4B). The CD163-positive M2 macrophages were more abundant in MCT-1-promoted tumors but were less in shMCT-1 tumors compared with the scramble control tumors (Fig. 3d), demonstrating MCT-1 impacts on the recruitment and polarization of TAMs. Further surveillance of THP-1 polarity in coculture with the MCT-1-overexpressed cells showed that the M2 markers (CD163 and CD206) were upregulated than co-cultured with control MDA-MB-231 cells or RPMI medium (Fig. 3e). However, the M2-like polarization was reduced by coculture with shMCT-1#3 cells relative to scramble shRNA-treated cells or RPMI medium (Fig. 3f), as reflected in CD163 and CD206 reduction. Upon coculture with shMCT-1#3 cells, THP-1 cells were preferentially polarized into pan-macrophages and M1-like macrophages (Fig. 3g), in which F4/80 and CD86 were significantly activated. Consistently, the M2 markers Arginase-1 and IL-10 in the polarized THP-1 cells were more induced after priming by the MCT-1-overexpressing cells (THP-1/MCT-1) than after priming by control cells (THP-1/control) (Fig. 3h), but Arginase-1 and IL-10 were greatly reduced in the THP-1 cells while cocultured with shMCT-1#3 cells (THP-1/shMCT-1) than with scramble control cells (THP-1/scramble).

Furthermore, when MDA-MB-231 (control vs, MCT-1) cells were pretreated with an IL-6R mAb (tocilizumab) and then co-cultured with THP-1 cells (Fig. 3I), the CD86 expression was induced in the polarized M1-like macrophages. However, the IL-6 pretreatment did not significantly increase CD86-positived M1 macrophage differentiation. In addition, the M2-like polarization revealed by CD163 expression was induced when THP-1 cells were cocultured with the IL-6-treated cancer cells but was inhibited when THP-1 cells were cocultured with tocilizumab-treated cancer cells (Fig. 3j). Accordingly, MCT-1-overexpressing breast cancer cells promotes M2-like macrophage polarization in vitro. Tocilizumab antagonizes the MCT-1 function to enhance M1 polarity, whereas IL-6 stimulates MCT-1 effect on M2 promotion.

### MCT-1/IL-6/IL-6R signaling mediates breast cancer stemness

**Fig. 4:**
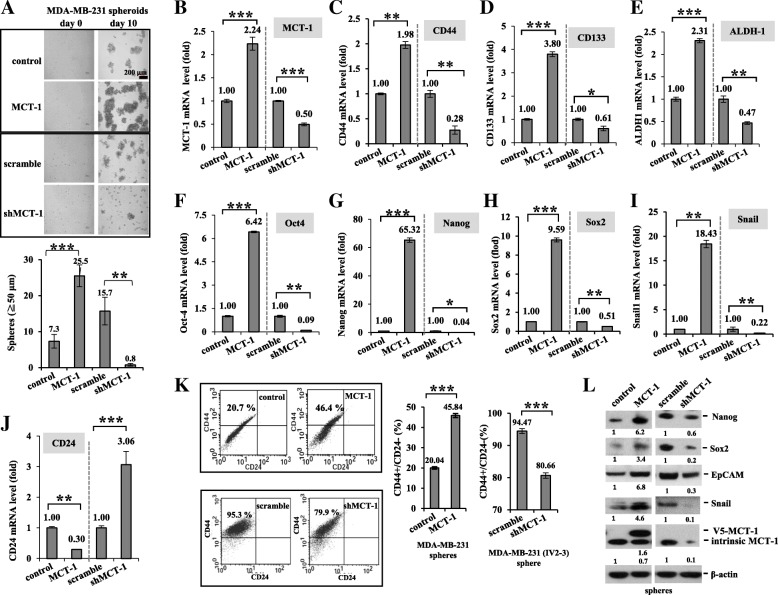
MCT-1 promotes MDA-MB-231 breast cancer stemness. (**a**) MDA-MB-231 sphere formation at day 10 was studied in different MCT-1 circumstances (control vs. MCT-1; scramble vs. shMCT-1#3). Mammospheroids (≧50 μm in diameter) were measured. The mRNA levels of the indicated gene were quantified by qRT-PCR in day 10–14 mammospheres. (**b**) MCT-1. (**c**) CD44. (**d**) CD133. (**e**) ALDH-1. (**f**) Oct4. (G) Nanog. (**h**) Sox2. (**i**) Snail. (**j**) CD24. (**k**) Flow cytometry was used to evaluate CD44(+)/CD24(−) subpopulations in the mammospheres. The results are expressed as the mean ± SD (*n* = 3). One-way ANOVA with a post hoc two-tailed t-test was used to calculate the statistical significance of pairwise comparisons. (*p < 0.05; **p < 0.01; *** *p* < 0.001) (**l**) Nanog, Sox2, EpCAM and Snail proteins were evaluated in the mammospheres. Relative protein amounts were compared with the controls. Different blots with normalized internal control were used to present the data of the experiment

IL-6/Stat3 signaling promotes breast cancer stemness [32]. Investigating BCSCs derived from MDA-MB-231 cells, we first identified that MCT-1 overexpression promoted MDA-MB-231 mammospheriods (3.49-fold) but that were significantly reduced after MCT-1 depletion in IV2–3 (shMCT-1#3) (0.05-fold) compared with that of the control and the scramble groups (Fig. 4a). Cancer stemness marker CD44 level was also elevated through MCT-1 induction (1.98-fold) (Fig. 4b) but decreased upon MCT-1 knockdown (0.28-fold) than the controls in mammospheroids as detected by qRT-PCR (Fig. 4c). Similarly, MCT-1 promoted CD133 (Fig. 4d), ALDH-1 (Fig. 4e), Oct4 (Fig. 4f), Nanog (Fig. 4g), Sox2 (Fig. 4h) and Snail (Fig. 4i) mRNA levels in the mammospheres, but those were all greatly reduced in shMCT-1#3. Conversely, CD24 expression was increased in shMCT-1#3 mammospheres (3.06-fold) but suppressed upon MCT-1 induction (0.3-fold) (Fig. 4j). Analyzing the mammospheres by flow cytometry, the CD24(−)/CD44(+) subpopulations were more abundant with overexpressed MCT-1 (46.4%) than the controls (20.7%) (Fig. 4k). Contrarily, the abundant CD24(−)/CD44(+) subpopulations in the high metastatic IV2–3 mammospheres (scramble, 95.3%) were decreased by shMCT-1#3 (79.9%). Because the MDA-MB-231 (IV2–3) subline has been selected from two rounds of lung metastasis; thus, it enriched higher cancer stem cells (95.3%) than the MCT-1-overexpressing cells (46.4%). Consistently, Nanog (Fig. 4l), Sox2, EpCAM and Snail proteins were induced by MCT-1 but suppressed in shMCT-1#3.

Also, we found that IL-6 treatment indeed further stimulated MDA-MB-231 mammosphere formation (Additional file 5: Figure S5A) along with increase of MCT-1 (Additional file 5: Figure S5B) CD44 (Additional file 5: Figure S5C), CD133 (Additional file 5: Figure S5D), ALDH-1 (Additional file 5: Figure S5E), Oct-4 (Additional file 5: Figure S5F), Sox2 (Additional file 5: Figure S5G) and Nanog (Additional file 5: Fig. 5h) mRNAs, particularly in an oncogenic MCT-1 background. Hence, IL-6 and MCT-1 collaboratively advance cancer stemness.

**Fig. 5:**
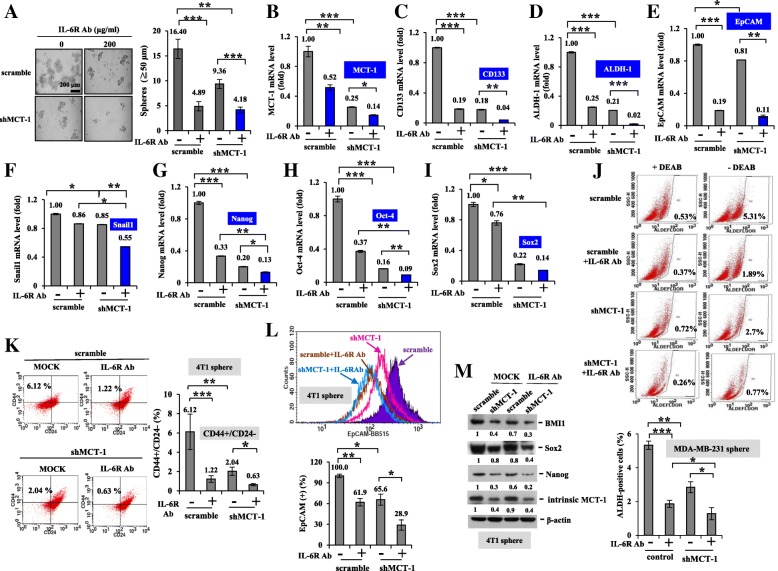
Tocilizumab and shMCT-1 synergistically inhibit breast cancer stemness. (**a**) The mammospheres derived from IV2–3 subline (scramble vs. shMCT-1#3) were evaluated upon tocilizumab (200 μg/ml) treatment for 20 days. The mRNA levels of the indicated gene were examined in MDA-MB-231 mammospheres and after tocilizumab challenge. (**b**) MCT-1. (**c**) CD133. (D) ALDH-1. (**e**) EpCAMP. (**f**) Snail. (**g**) Nanog. (H) Oct-4. (**i**) Sox2. (**j**) ALDH(+) cancer stem cells were detected by an ALDEFLUOR assay as DEAB inhibited ALDH activity, to accurately analyze ALDH activity in day 14 mammospheres and after tocilizumab treatment. (**k**) CD44-FITC and CD24-Alexa staining identified CD24(−)/CD44(+) subpopulations in the 4 T1 mammospheres (scramble and shMCT-1#3–28) and upon tocilizumab treatment for 6 days. (**l**) EpCAM(+) cancer stem cells were evaluated in the 4 T1 mammospheres and upon tocilizumab treatment for 6 days using EpCAM-BB515 staining. The results are expressed as the mean ± SD (*n* = 3). One-way ANOVA with a post hoc two-tailed t-test was used to calculate the statistical significance of pairwise comparisons. (*p < 0.05; **p < 0.01; *** *p* < 0.001) (**m**) The cancer stemness molecules in scramble and shMCT-1#3–28 cells were examined before and after tocilizumab treatment

Consistent with an IL-6R increase (Fig. 2e), IL-6R mRNA levels were elevated by MCT-1 but inhibited by shMCT-1#3 in mammospheres (Additional file 5: Figure S5I). Tocilizumab (IL-6R Ab) treatment suppressed MCT-1-induced mammosphere formation (Additional file 5: Figure S5J) combined with decrease of IL-6R (Additional file 5: Figure S5K), IL-6 (Additional file 5: Figure S5L), CD44 (Additional file 5: Figure S5M), ALDH-1 (Additional file 5: Figure S5N), EpCAM (Additional file 5: Figure S5O) and Oct-4 (Additional file 5: Figure S5P) mRNAs. Moreover, tocilizumab repressed the MCT-1-induced CD44(+)/CD24(−) subpopulations (55.7%) to the control degree (32.5%) (Additional file 5: Figure S5Q), confirming that IL-6R immunotherapy inhibited MCT-1-promoted cancer stemness.

Importantly, tocilizumab (IL-6R Ab) treatment further inhibited the MDA-MB-231 mammospheroids (Fig. 5a) as well as levels of MCT-1 (Fig. 5b), CD133 (Fig. 5c), ALDH-1 (Fig. 5d), EpCAM (Fig. 5e), Snail (Fig. 5f), Nanog (Fig. 5g), Oct-4 (Fig. 5h) and Sox2 (Fig. 5i) mRNAs in the shMCT-1#3 cellular background. These results indicate that MCT-1 induces cancer stemness via multiple ways in addition to IL-6R. MCT-1 and IL-6/IL-6R pathway can together advance cancer stemness effects. As aldehyde dehydrogenase (ALDH) activity reduced by diemethylamino-benzaldehyde (DEAB) (Fig. 5j), it specifically inhibited ALDH to accurately identify the ALDH(+) cancer stem cells in each group. The ALDH(+) cells were found to be fewer in the shMCT-1#3 group (2.7%) than in the scramble set (5.31%), and the ALDH(+) cells in the shMCT-1 condition were further suppressed by tocilizumab (0.77%).

Similarly, tocilizumab treatment of 4 T1 cells further repressed MCT-1 (Additional file 6: Figure S6A), CD133 (Additional file 6: Figure S6B), Snail (Additional file 6: Figure S6C), Nanog (Additional file 6: Figure S6D) and Oct-4 (Additional file 6: Figure S6E) mRNA levels, particularly in shMCT-1 mammospheres. Furthermore, shMCT-1 (#3–28) reduced the CD24(−)/CD44(+) subpopulations (2.04%) than that identified in the scramble set (6.12%) of the 4 T1 mammospheres (Fig. 5k), and tocilizumab further reduce the CD24(−)/CD44(+) subpopulations in the shMCT-1 (#3–28) background (0.63%). Using EpCAM to define 4 T1 cancer stemness (Fig. 5l), EpCAM(+) cells were reduced in shMCT-1 (#3–28) mammospheres (65.6%) than in the scramble control (100%), and tocilizumab further decreased the EpCAM(+) populations (28.9%) in the shMCT-1 background. In consistence, tocilizumab further decreased BMI-1 (Fig. 5m), Sox2 and Nanog amounts in the shMCT-1 (#3–28) mammospheres. Consequently, IL-6R immunotherapy inhibits the oncogenicity of MCT-1 and cooperates with MCT-1 knockdown to profoundly suppress cancer stemness.

### miR-34a inhibits MCT-1-promoted IL-6R expression and M2 polarization

MicroRNAs (miRNAs) communicate between tumor cells and their microenvironment; thus, the miRNAs are used for anti-cancer therapy/regimens [33]. To identify global microRNA profiling in the MCT-1 pathway, RNA samples isolated from the tumors of TNBC cells (MDA-MB-468) [18] were subjected to the direct, digital counting of miRNA levels by an nCounter miRNA Expression Assay (NanoString Technologies), as described before [34]. Screening of the 800-miRNA panel revealed 21 downregulated miRNAs and 43 upregulated miRNAs in the shMCT-1 tumor compared with the scramble group (Additional file 7: Figure S7A). The levels of miRNAs altered in the shMCT-1 tumor are listed (Additional file 7: Figure S7B). Among the upregulated 43 miRNAs, the tumor-suppressive miRNAs (miR-34a, miR-99a, miR-125b) were increased in shMCT-1 (#3) of IV2–3 subline as detected by qRT-PCR analysis (Additional file 7: Figure S7C and D), showing that shMCT-1 induced the tumor suppressor miRNAs.

**Fig. 6:**
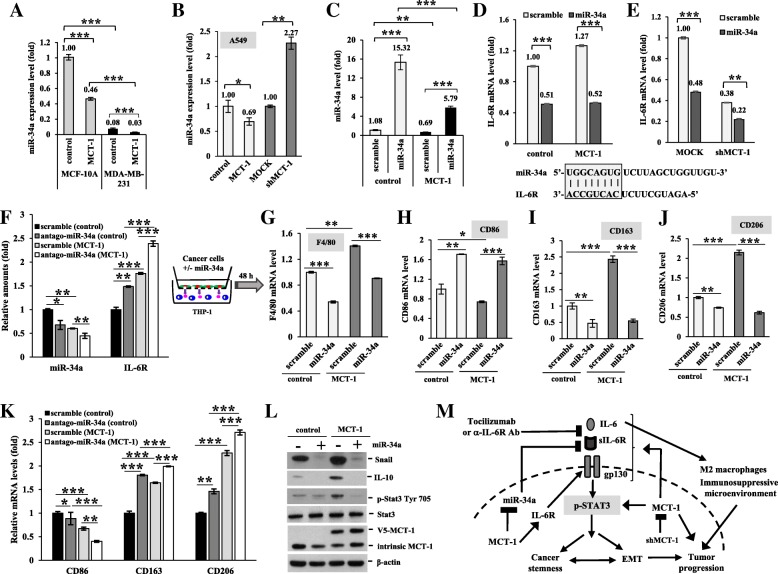
miR-34a suppresses IL-6R expression and M2-like macrophage polarization. (**a** and **b**) MiR-34a levels were examined by qRT-PCR analysis in MCF-10A, MDA-MB-231 and A549 cells with different MCT-1 expression conditions. (**c**) The mature miR-34a levels were compared in the MDA-MB-231 cells (control vs. MCT-1) after transfection of pre-miR-34a and scramble-miR oligonucleotides. (**d** and **e**) IL-6R mRNA levels were compared in different MCT-1 backgrounds (control vs. MCT-1; scramble vs. shMCT-1#3) after transfection of pre-miR-34a and scramble-miR oligonucleotides. (**f**) IL-6R mRNA levels were compared in different MCT-1 backgrounds (control vs. MCT-1; scramble vs. shMCT-1#3) after transfection of pLemiR-antago-miR-34a or pLemiR-scramble. The MDA-MB-231 cells were virally transfected with pLemiR-scramble or pLemiR-pre-miR-34a, and then were co-incubated with THP-1 for 48 h to analyze the macrophage differentiation markers. (**g**) Pan-macrophages (indicated by F4/80). (**h**) M1-like macrophages (shown by CD86). (**i** and **j**) M2-like macrophages (revealed by CD163 and CD206). The results are expressed as the mean ± SD (*n* = 3). One-way ANOVA with a post hoc two-tailed t-test was used to calculate the statistical significance of pairwise comparisons. (*p < 0.05; **p < 0.01; *** *p* < 0.001). (**k**) THP-1 differentiation into M1 (CD86) or M2 (CD163 and CD206) macrophages were examined by qRT-PCR after culture with the MDA-MB-231 cells (control vs. MCT-1) introduced with pLemiR-antago-miR-34a or pLemiR-scramble. (**l**) The amounts of Snail, IL-10 and Stat3 were inspected in the THP-1 cells. Different blots with normalized internal control were used to present the data of the experiment. (**m**) Implications of MCT-1-targeted therapies. The oncogenic MCT-1 pathway in TNBC can be retracted by MCT-1 inhibitor(s), IL-6R immunotherapy or miR-34a expression, together they can further prevent EMT progression, cancer stemness, M2 macrophage polarization and tumor progression

We found that miR-34a levels were more abundant in MCF-10A cells than in MDA-MB-231 cells but significantly repressed upon MCT-1 overexpression (Fig. 6a). Intriguingly, MCT-1 depletion in IV2–3 subline (p53-mutant) restored the miR-34a level independent of p53 function (Additional file 7: Figure S7D) similar to that of invasive lung cancer A549 cells (p53-wildtype) upon MCT-1 knockdown (Fig. 6b). Even after introducing pre-miR-34a oligonucleotides into MDA-MB-231 cells (Fig. 6c), the mature miR-34a levels were still much suppressed in the MCT-1-overexpressing cells (5.79-fold) than in control cells (15.32-fold). The IL-6R/STAT3/miR-34a loop mediates cancer invasion and metastasis [8]. Upon miR-34a re-expression, the MCT-1-induced IL-6R expression was decreased (Fig. 6d) and IL-6R was further abrogated in shMCT-1#3 cells (Fig. 6e). These indicate that miR-34a and shMCT-1 can synergistically inhibit IL-6R function. To further investigate whether inhibition of miR-34a induces IL-6R expression in MCT-1 pathway, we found that antago-miR-34a transfection into the cells effectively suppressed miR-34a level that further promoted IL-6R in MCT-1 overexpression background (Fig. 6f), comparing with scramble-miR transfectant. Because overexpressing MCT-1 in a loss-of-function miR34 condition still highly induces IL-6R, MCT-1 promotes IL-6R at least in part independently of miR-34.

To explore the miR-34a role in macrophage differentiation, MDA-MB-231 cells were virally transduced with pLemiR-scramble-miR (scramble) or with pLemiR-pre-miR-34a (miR-34a) followed by priming THP-1 cells. The miR-34a-expressed cancer cells more induced THP-1 polarization into M1-like CD86-positive macrophages than that of the scramble cells (Fig. 6h). Inversely, miR-34a induction suppressed the polarization of pan-macrophages (F4/80) (Fig. 6g) and M2-like macrophages promoted by MCT-1, as characterized by decrease in CD163 (Fig. 6i) and CD206 (Fig. 6j). However, M2 macrophage markers (CD163 and CD206) were advanced and M1 macrophage marker (CD86) was suppressed in THP-1 cells after co-culture with the cancer cells transfected with antago-miR-34a compared with scramble-miR transfectant (Fig. 6k). Since CD163 and CD206 expression are constitutively activated while overexpressing MCT-1 in a loss-of-function miR34 condition, MCT-1 also promotes the M2 markers independent of miR-34 pathway.

Similarly, the inducers of M2 differentiation (Snail, IL-10 and phospho-active Stat3) which were promoted by MCT-1 were significantly reduced upon miR-34a expression (Fig. 6l). Hence, miR-34a expression in TNBC cells mediates M1 polarization but antago-miR-34a promotes M2 plasticity.

Collectively, oncogenic MCT-1 activation stimulates the IL-6/IL-6R/Stat3 axis that enhances EMT progression (Fig. 6m), cancer stemness and M2 polarization in the TNBC system. IL-6 enhanced the effect of MCT-1 on EMT plasticity, M2 polarity and cancer stemness, which were suppressed by tocilizumab. The combination of MCT-1 and IL-6/IL-6R pathway cooperatively enhanced cancer stemness and the oncogenic effects. Furthermore, targeting MCT-1 induced miR-34a that may reprogram EMT and macrophage plasticity and inhibit TNBC stemness and tumor progression. Therefore, MCT-1 inhibition combined with IL-6R antagonist or miR-34a expression may further renovate non-BCSC effect and tumor-suppressive M1 macrophages in TNBC.

## Discussion

Inflammatory microenvironment plays an important role for cancer progression [35]. Immunotherapy is emerging as a novel promising strategy for TNBC treatment [36]. The TNBC cells with MCT-1 overexpression secrete more inflammatory cytokines IL-6, MCP-1 and GM-CSF. GM-CSF and IL-6 influences TAM polarity, and MCP-1 functionally promotes osteoclast differentiation from monocytes [28, 37, 38]. Moreover, IL-6 and MCP-1/CCL2 regulate the fate of CSCs and the TME [39]. Therefore, immunotherapeutic interventions of the cytokine pathways may modify the TME and eradicate CSCs.

Serum IL-6 levels are increased with advanced stages and related to poor survival in various cancers [40], and IL-6 drives breast cancer metastasis and stemness [41]. IL-6/IL-6R antagonists as anti-breast cancer agents have not been broadly investigated and are even less studied in TNBC. Tocilizumab, a recent FDA-approved humanized mAb used to treat autoimmune and inflammatory diseases, has been proposed to inhibit the trastuzumab-resistant HER2(+) breast cancer [42].

Abundant IL-6 released from aggressive cancer cells stimulates angiogenesis and tumor evasion from immune surveillance [43]. Nevertheless, IL-6 promotes antitumor effect by boosting T-cell immunity and by trafficking antitumor T cells to lymph nodes and tumor sites, executing the cytotoxic effects. It is unclear whether MCT-1 also influences Th1-Th2 polarization. Reflecting the Th1-Th2 polarization of T cells [44], the activation of M1 (pro-inflammatory) and M2 (anti-inflammatory) macrophages are functionally modified by Th1 and Th2 cytokines. Th1 cytokines such as IFN-γ and GM-CSF induce M1 polarization, which produces pro-inflammatory cytokines (IL-1β, IL-6, IL-12, IL-23 and TNF-α). Th2 cytokines such as IL-4 and IL-13 promote M2 polarization, which produces anti-inflammatory cytokines (IL-10 and TGF-β).

YY1 transcriptionally activates IL-6 gene expression [45], and the EGFR signaling triggers IL-6 production via NF-kB activation [46]. Oncogenic MCT-1 activation promotes the expression of YY1 and EGFR [23], suggesting that MCT-1 may increase IL-6 expression via the YY1-EGFR signaling amplification. NOTCH activation through NO facilitates constitutive IL-6-dependent STAT3 activation [32], promoting breast cancer stemness. MCT-1 stimulated IL-6/Stat3 signaling (Fig. 2e), suggesting that MCT-1 may also stimulate the NO/NOTCH pathway to mediate breast cancer metastasis and recurrence*.* In addition, systematic administration of IL-6/IL-6R antagonist(s) with MCT-1 inhibitor(s) may promote immune cell infiltration to advance therapeutics against tumor heterogeneity and aggressiveness, with fewer adverse effect(s).

MCT-1 induces PD-L1 but reduces miR-34a. Targeting PD-L1 by miR-34a in the cancer cells prevent the PD-1/PD-L1 interaction that increases anti-tumor activity [47, 48]. miR-34a inhibits cancer stemness via targeting CD44 [49]; miR-34a expression inhibits TGF-β-induced EMT and downregulates Snail [50], Slug and ZEB1 as well as the stemness factors (BMI1, CD44, CD133, OLFM4 and c-MYC). Reciprocally, Snail and ZEB1 repress the miR-34a function to promote EMT [50, 51]. To sustain the immune escape mechanism, the TME recruits and changes myeloid cells to TAMs [52], dendritic cells, myeloid-derived suppressor cells and neutrophils. Macrophage colony-stimulating factor (M-CSF) induces M2 polarization [53], and miR-34a targets receptor of M-CSF, which regulates dendritic cell maturation to maintain a proper immune balance in anti-Th2 response, immune stimulation and tumor resistance. We now identify that miR-34a expression in p53-mutant TNBC cells promotes M1 polarization, emphasizing that miR-34a potentially modifies the tumor immunity and heterogenicity. MCT-1 antagonist combined with miR-34a expression may alter the polarity and activation of the immune cells, thus improving the efficacy of TNBC treatment.

## Conclusions

MCT-1/miR-34a/IL-6/IL-6R is a novel signaling axis identified in TNBC. MCT-1 inhibition combined with IL-6/IL-6R immunotherapy or with miR-34a expression would be a new stratagem for administration of TNBC. Better understanding the circuits between cytokines and microRNAs orchestrated by the oncogenic activity will facilitate breast cancer diagnosis, prevention and therapeutics.

## Methods

### THP-1 polarization and cancer cell invasion

Cancer cells (1 × 10^5^) were seeded into the upper chamber of Falcon® Cell Culture Inserts (Corning, Corning, NY) and cocultured with THP-1 monocytes (1 × 10^6^) in the bottom chamber for 48 h. A control experiment was conducted as THP-1 cells co-incubated with RPMI medium alone. The markers of pan-macrophages (F4/80), M1 macrophage (CD86) and M2 macrophages (CD163 and CD206) were analyzed in the primed THP-1 cells by qRT-PCR using the synthesized primers (MDBio) listed in Additional file 8: Table S1.

Moreover, MDA-MB-231 cells (1 × 10^5^) were pretreated with IL-6 (50 ng/ml), IL-6R mAb (0.5 μg/ml) (PeproTech, Rocky Hill, NJ) or tocilizumab **(**150 μg/ml) (CHUGAI, Tochigi, Japan) for 24 h; next, the pretreated MDA-MB-231 cells (2 × 10^4^) were used to prime THP-1 cells (1 × 10^6^) in Cell Culture Inserts for 48 h. The preconditioned THP-1 cells with 10% FBS/RPMI or the medium alone were placed into the lower chamber of Cell Invasion Inserts (Corning), and the invasiveness of serum-free parental MDA-MB-231 cells (2 × 10^4^) in the upper chamber were analyzed for 24 h. Supplementary methods can be found in Additional file 9.

### Cytokine array analysis

Cells were seeded in 10 cm plates (1 × 10^6^ cells/plate) with serum-free RPMI for 24 h. The condition medium (CM) was centrifuged for 20 min at 1000 x g at 4 °C and then collected the supernatant to perform the assay. Human cytokine array C6 membranes (RayBiotech, Norcross, GA) were incubated overnight with 1 ml of CM at 4 °C. After washing with the buffer, the membranes were incubated overnight with biotin-conjugated Abs (human cytokine antibody cocktail), washed thrice, reacted with horseradish peroxidase-conjugated streptavidin in blocking buffer for 2 h and performed photography on X-ray film.

### Analysis of the secretory IL-6 and IL-6R

Human IL-6 and IL-6R ELISA MAX™ Deluxe (BioLegend, San Diego, CA) analyzed the secreted IL-6 amounts. Briefly, the indicated cells were seeded in 6-well plates (2 × 10^5^ cells/well) with serum-free RPMI medium for 24 h. The condition medium was centrifuged for 20 min at 1000×g at 4 °C and then collected the supernatant to carry out the assay. The ELISA plates were coated with the diluted IL-6 or IL-6R A, incubated overnight at 4 °C, washed 4 times with the buffer (0.05% Tween-20 in PBS) and incubated with the diluted buffer (included in the kit) for 1 h. After washing for 4 times, the plates were incubated with the diluted standards (included in the kit) and the 100 μl conditioned medium for 2 h, rinsed 4 times, incubated with the diluted detection Ab (included in the kit) for 1 h, washed 5 times and incubated with 3, 3′, 5, 5′-Tetramethylbenzidine substrate solution (included in the kit) in the dark for 15 min. Reactions were stopped and detected the absorbance at 450 nm within 15 min. The results were normalized with the numbers of the seeded cancer cells.

### Cancer stemnness analysis

A single-cell suspension was cultured on 6-well ultralow-attachment plate (Corning) at a density of 4 × 10^4^ cells/well in serum-free DMEM/F12 medium with 1% L-glutamine, 1% penicillin/streptomycin, 2% B27 (Invitrogen), 20 ng/ml EGF (Sigma-Aldrich) and 20 ng/ml FGFb (PeproTech). IL-6 (50 μg/ml) (PeproTech) or tocilizumab (200 μg/ml) was added to examine the mammosphere formation. Mammospheroids were photographed at a magnification of 200× using a Nikon DIAPHOT300 microscope at the indicated time. Mammosphere cells (1 × 10^5^) were further stained with anti-human CD24-PE (BD Pharmingen), anti-human CD44-FITC (BD Pharmingen), CD24-Alexa 647 (BD Pharmingen) or EpCAM-BB515 (BD Pharmingen) for 1 h at 4 °C, PBS rinsed and resuspended in 500 μl PBS. CD44-FITC and EpCAM-BB515 were excited at 490 nm, and the emissions were determined by FL1 PMT (515–545 nm bandpass filter). CD24-Alexa 647 was excited at 633 nm, and the emissions were determined by FL-4 PMT (653–669 nm bandpass filter). BD FACSCalibur flow cytometry (BD Bioscience) and Cell Quest software (BD Biosciences) were used to identify CD44(+)/CD24(−) and EpCAM(+) subpopulations.

### Tumor progression

Six to eight week-old female BALB/c nude mice (BALB/cAnN.Cg-Foxn1^nu^/CrlNarl) were purchased from the National Laboratory Animal Center (Taipei, Taiwan), according to the Animal Use Protocol approved by the National Health Research Institutes (NHRI-IACUC-106012-A). MDA-MB-231 cells (1 × 10^5^) or MDA-MB-231 (IV2–3) cells (1 × 10^6^) bearing a pcDNA3.1-luciferase reporter were injected into the fourth mammary fat pads of the mice for a tumor progression study. Luminescent tumor images were checked weekly after the intraperitoneal injection of luciferin (150 mg/kg) (PerkinElmer, Waltham, MA) for 10 min and detected by a Xenogen IVIS 200 bioluminescence imaging system (Caliper LifeSciences, Hopkinton, MA).

### Clinical study

Human breast tissue microarrays (BR1503c, BR953 and BRN801a) were obtained from US Biomax (Rockville, MD), the TNBC tissue microarray was obtained from Pantomics (BRC964) (Richmond, CA) and the adjacent normal breast tissue microarray was obtained from SOBC (Hbre-Duc052Bch-01) (Pudong, Shanghai, China). Samples were stained with MCT-1 Ab (1:200, GeneTex, GTX117793) using a Discovery XT Automated IHC/ISH Slide Staining System (Ventana Medical System, Tucson, AZ) and an UltraView Universal DAB Detection Kit (Ventana Medical System). The results were classified according to the clinical and pathology information provided by the companies.

The MCT-1, IL-6 and IL-6R mRNA expression levels in breast carcinomas versus normal breast tissues were analyzed in the Oncomine database (http://www.oncomine.org). The Kaplan-Meier Plotter (http://www.kmplot.com) analyzed the probability of OS and RFS in breast cancer patients. The survival curve was exported using GraphPad Prism software. MCT-1, IL-6 and IL-6R mRNA levels were quantified by using TissueScan Breast Cancer Tissue qPCR Panels (I, III and IV) (OriGene Technologies, Inc., Rockville, MD). Relative mRNA levels were calculated: ΔΔCT = ΔCt cancer-ΔCt normal tissue. The fold change in the gene was calculated using the formula 2^−ΔΔCT.^

### MicroRNA profiling

The nCounter® Human v2 miRNA Panel (nanoString, Seattle, WA) containing 800 unique miRNA barcodes was used. RNA samples were extracted from the MDA-MB-468 tumors (MOCK vs. shMCT-1) by TRIzol™ reagent (Invitrogen). Total RNAs (100 ng) were used as input for the nanoString platform. Mature miRNAs were multiplied by annealing to a human-specific tag sequence (miRtag) via melting-temperature-controlled splinted ligation onto the 3′-end. Excess unligated miRtags were then removed by enzymatic purification, and the resulting material was hybridized at 65 °C for 16 h with a panel of miR: tag-specific nCounter capture and barcoded reporter probes. The raw data were normalized by 6 positive-control and 8 negative-control probe pairs. All the samples were analyzed in triplicate. The miRNA amounts were quantified by a nanoString nCounter Digital Analyzer and gene-expression system.

### Expression of miR-34a and antago-miR-34a

Scramble-miR and pre-miR-34a oligonucleotides (Biotools, New Taipei City, Taiwan) were transiently transfected into cells using Lipofectamine 3000 (Thermo Fisher Scientific) for 48 h. The pre-miR-34a was cloned from pcDNA3-miR-34a using the primers (forward: 5′-ggctcgagTAGTTGCCTGGG CTGGTCTT; reverse: 5′-gggcggccgcCCTGTGCCTTTTTCCTTCC). The thermal cycling conditions were conducted at 94 °C for 2 min, followed by 35 cycles at 94 °C for 15 s, 55 °C for 1 min and 68 °C for 1 min. The amplicon was constructed into the Xho*I* and Not*I* sites of a pLemiR-NS (nonspecific hairpin) vector (Open Biosystem, Huntsville, AL, USA). The scramble-miR was also constructed into a pLemiR-NS vector. The 293 T cells were transfected with pLemiR-pre-miR-34a and pLemiR-scramble-miR using the TransIT-LT1 Transfection Reagent (Mirus Bio LLC, Madison, WI). The lentiviral supernatants were infected the indicated cells.

The antago-miR-34a was constructed by the primers (forward: 5′-gatccTGGCCAGTGTCTTAGCTGGTTGTttcaagagaACAACCAGCTAAGACACTGGCCAttttt; reverse:5′-agcttaaaaaTGGCCAGTGTCTTAGCTGGTTGTtctcttgaaACAACCAGCTAAGACACTGGCCA). The primers were annealed in the buffer (0.1 M potassium acetate, 30 mM HEPES KOH, and 2 mM magnesium acetate) at 95 °C for 2 min and then cool down to 25 °C within 50 min. The products were cloned into the BamH*I* and Hind*III* sites of a pRNA-U6.1 vector (GeneScript, Piscataway, NJ) and confirmed by DNA sequencing. Cells were transfected with Lipofectamine 3000 reagent (Thermo Fisher Scientific) according to the manufacturer’s protocol. 

## Additional files

Additional file 1: **Figure S1.** MCT-1 is a prognostic marker of human breast carcinoma. (PDF 215 kb)

Additional file 2: **Figure S2.** MCT-1 overexpression increases size of the MCF-10A acini. (PDF 223 kb)

Additional file 3: **Figure S3.** MCT-1 expression regulates MMP activity and breast cancer cell movements. (PDF 260 kb)

Additional file 4: **Figure S4.** CD80-positive M1 macrophages enriched in the TME after MCT-1 knockdown. (PDF 289 kb)

Additional file 5: **Figure S5.** MCT-1/IL-6/IL-6R signaling promotes breast cancer stemness. (PDF 259 kb)

Additional file 6: **Figure S6.** Targeting MCT-1 and tocilizumab treatment together suppress 4 T1 cancer stemness. (PDF 144 kb)

Additional file 7: **Figure S7.** MicroRNA profiling in the MCT-1 pathway. (PDF 48 kb)

Additional file 8: **Table S1.** Primer sequences. (PDF 104 kb)

Additional file 9: Supplementary Methods. Cell culture. Plasmid construction and transfection. Targeting MCT-1 gene. Targeting IL-6 gene. Antibodies (Abs) and protein analysis. MCF-10A acinar morphogenesis. Cell invasion and migration assays. Gelatin zymography assay. Cell fractionation. Quantitative RT-PCR analysis of cancer stemness markers. ALDEFLUOR assay. Immunohistochemistry study. Quantification of miR-34a levels. Statistical analysis. (PDF 227 kb)

## Abbreviations

BCSCs: breast cancer stem cells
CM: conditioned medium
DCIS: ductal carcinoma in situ
DEAB: diemethylamino-benzaldehyde
ELISA: enzyme-linked immunosorbent assay
EMT: epithelial-mesenchymal transition
gp130: glycoprotein 130
IDC: invasive ductal carcinoma
IL-6: interleukin-6
IL-6R: IL-6 receptor
M-CSF: macrophage colony-stimulating factor
MCT-1: Multiple Copies in T-cell Malignancy 1
miR-34a: microRNA-34a
MMPs: matrix metalloproteinases
OS: overall survival
PD-L1: programmed death-ligand 1
qRT-PCR: quantitative RT-PCR
RFS: recurrence-free survival
sIL-6R: soluble IL-R
Stat3: activate signal transducer and activator of transcription 3
TAMs: tumor-associated macrophages
TIMs: tissue inhibitors of metalloproteinase inhibitors
TME: tumor microenvironment
TNBC: triple-negative breast cancer
