# Supplementary material for: MCT-1/miR-34a/IL-6/IL-6R signaling axis promotes EMT progression, cancer stemness and M2 macrophage polarization in triple-negative breast cancer
Source: Mol Cancer. 2019 Mar 18;18:42. doi: 10.1186/s12943-019-0988-0 (PMC6421700; doi:10.1186/s12943-019-0988-0)
Supplement: Supplementary file 1 — Figure S1. MCT-1 is a prognostic marker of human breast carcinoma. (PDF 215 kb) [file 12943_2019_988_MOESM1_ESM.pdf]

Fig. S1

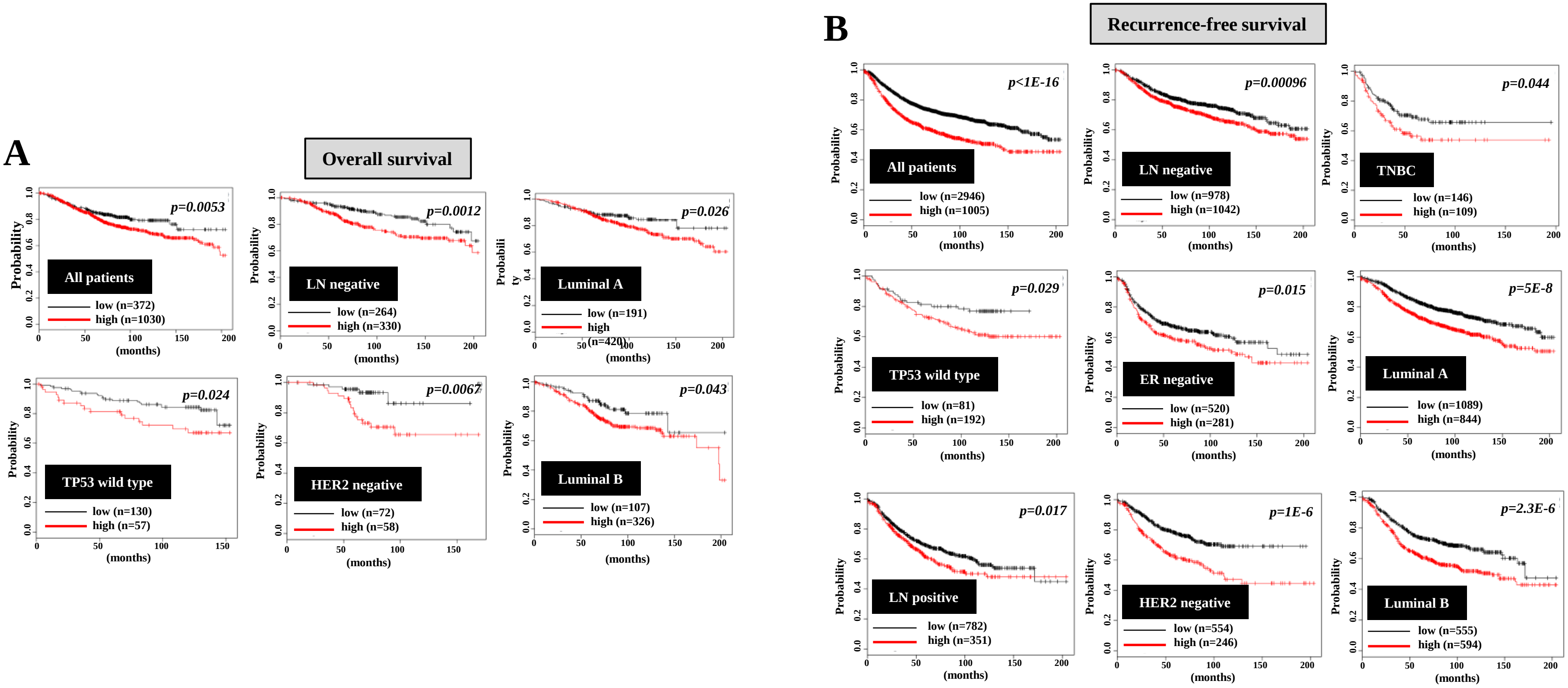

Supplementary Fig. S1. MCT-1 is a prognostic marker of human breast carcinoma. Using an analysis of the *Kaplan–Meier* plotter database, MCT-1 gene activation associated with OS **(A)** and RFS **(B)** were identified in different molecular types of breast cancer. A log rank test indicated the p-value.
