# Supplementary material for: MCT-1/miR-34a/IL-6/IL-6R signaling axis promotes EMT progression, cancer stemness and M2 macrophage polarization in triple-negative breast cancer
Source: Mol Cancer. 2019 Mar 18;18:42. doi: 10.1186/s12943-019-0988-0 (PMC6421700; doi:10.1186/s12943-019-0988-0)
Supplement: Supplementary file 2 — Figure S2. MCT-1 overexpression increases size of the MCF-10A acini. (PDF 223 kb) [file 12943_2019_988_MOESM2_ESM.pdf]

Fig. S2

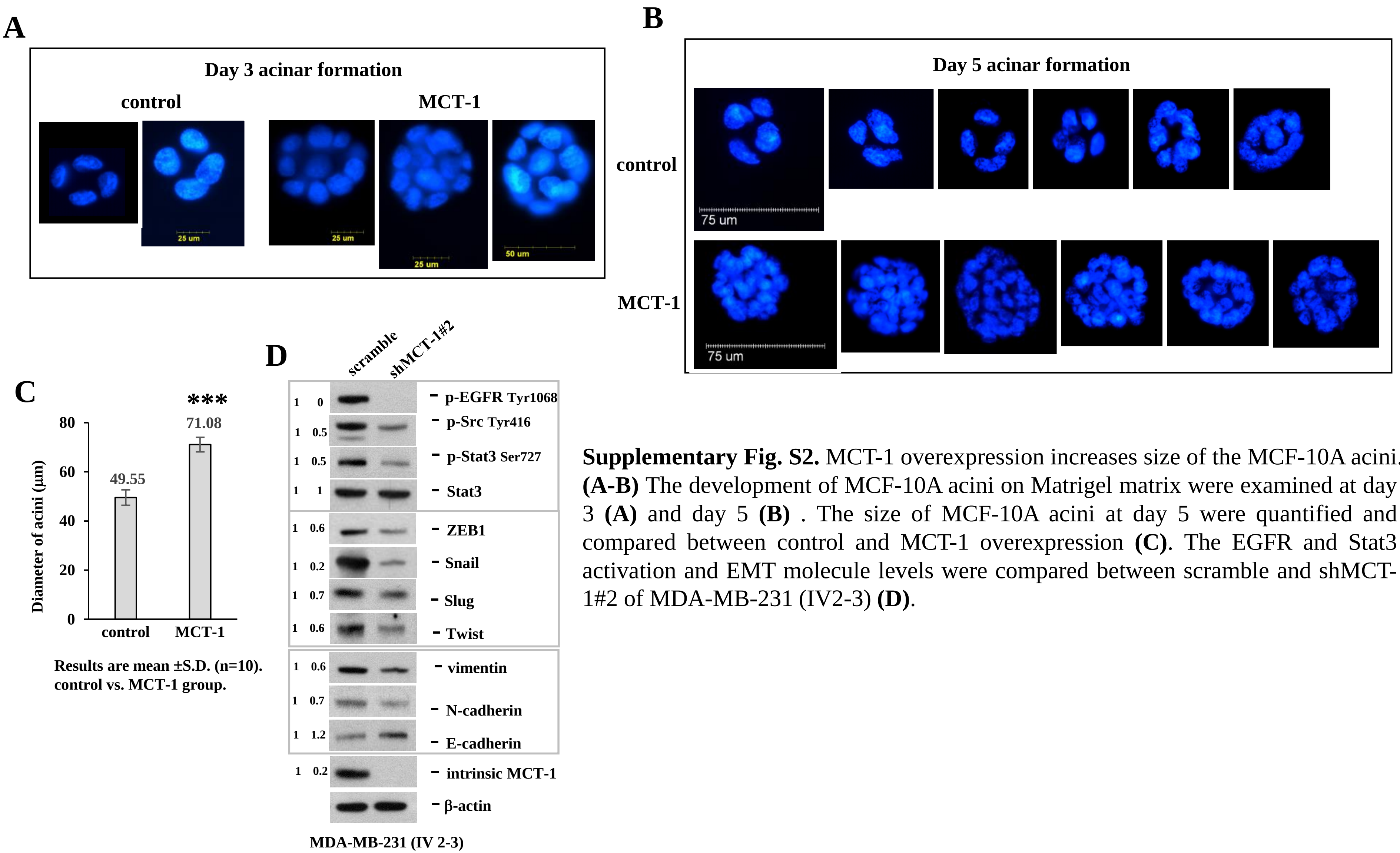

Supplementary Fig. S2. MCT-1 overexpression increases size of the MCF-10A acini. **(A-B)** The development of MCF-10A acini on Matrigel matrix were examined at day 3 **(A)** and day 5 **(B)** . The size of MCF-10A acini at day 5 were quantified and compared between control and MCT-1 overexpression **(C)**. The EGFR and Stat3 activation and EMT molecule levels were compared between scramble and shMCT-1#2 of MDA-MB-231 (IV2-3) **(D)**.
