# Supplementary figures and images for: MCT-1/miR-34a/IL-6/IL-6R signaling axis promotes EMT progression, cancer stemness and M2 macrophage polarization in triple-negative breast cancer
Source: Mol Cancer. 2019 Mar 18;18:42. doi: 10.1186/s12943-019-0988-0 (PMC6421700; doi:10.1186/s12943-019-0988-0)

**Fig. S3**

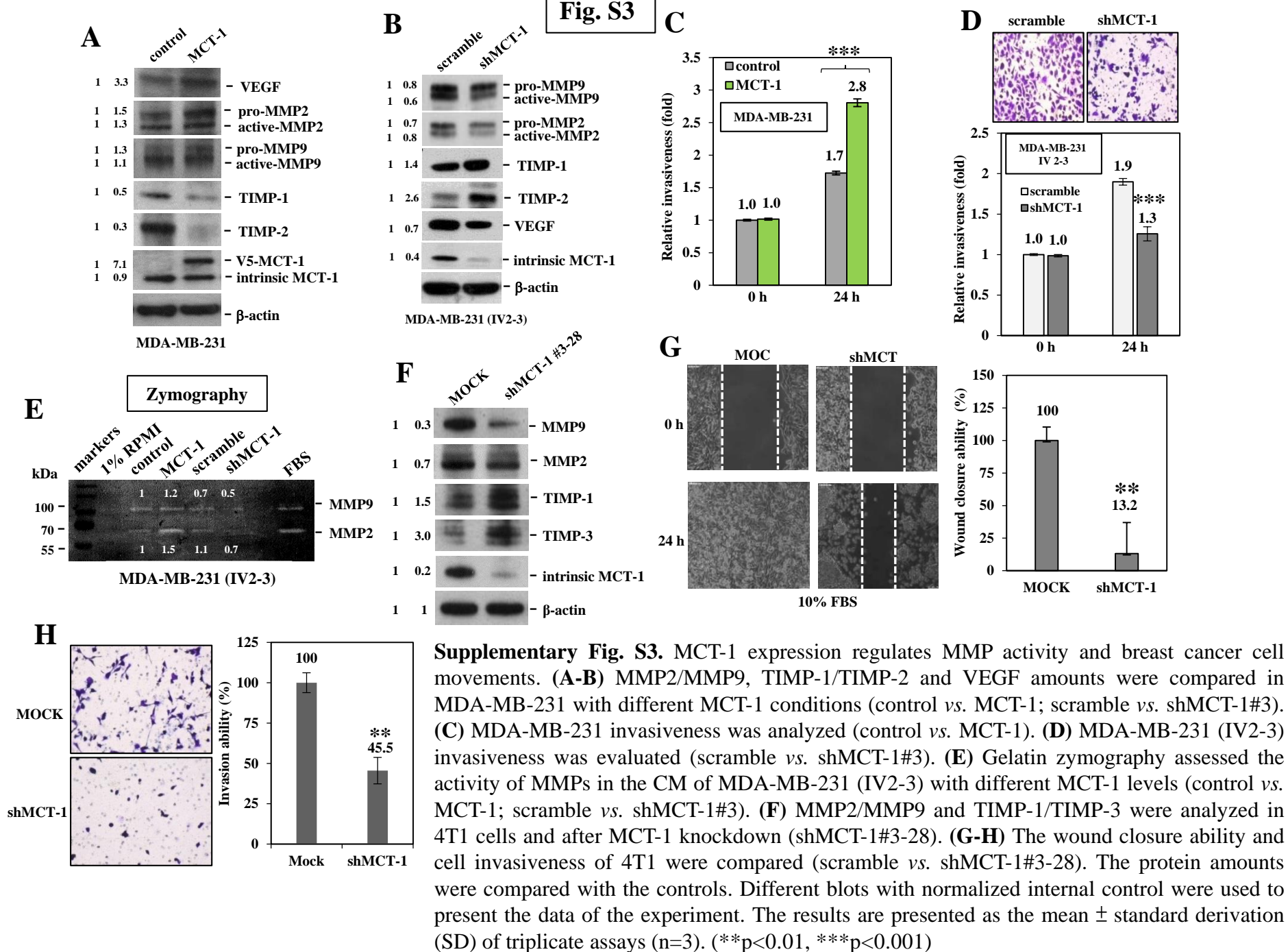

Supplement: Supplementary file 3 — Figure S3. MCT-1 expression regulates MMP activity and breast cancer cell movements. (PDF 260 kb) [file 12943_2019_988_MOESM3_ESM.pdf]

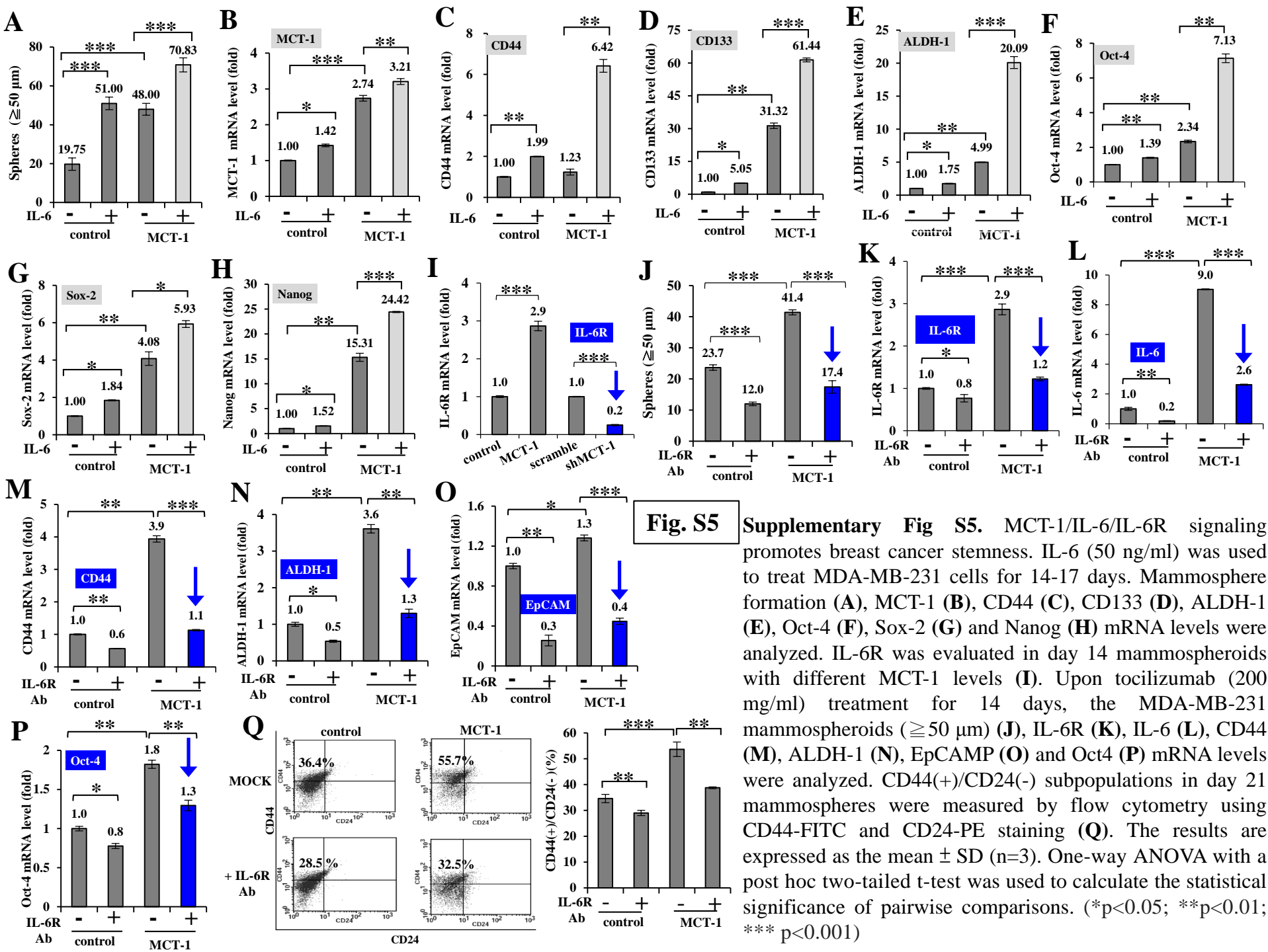

Supplement: Supplementary file 5 — Figure S5. MCT-1/IL-6/IL-6R signaling promotes breast cancer stemness. (PDF 259 kb) [file 12943_2019_988_MOESM5_ESM.pdf]
