# Supplementary material for: MCT-1/miR-34a/IL-6/IL-6R signaling axis promotes EMT progression, cancer stemness and M2 macrophage polarization in triple-negative breast cancer
Source: Mol Cancer. 2019 Mar 18;18:42. doi: 10.1186/s12943-019-0988-0 (PMC6421700; doi:10.1186/s12943-019-0988-0)
Supplement: Supplementary file 4 — Figure S4. CD80-positive M1 macrophages enriched in the TME after MCT-1 knockdown. (PDF 289 kb) [file 12943_2019_988_MOESM4_ESM.pdf]

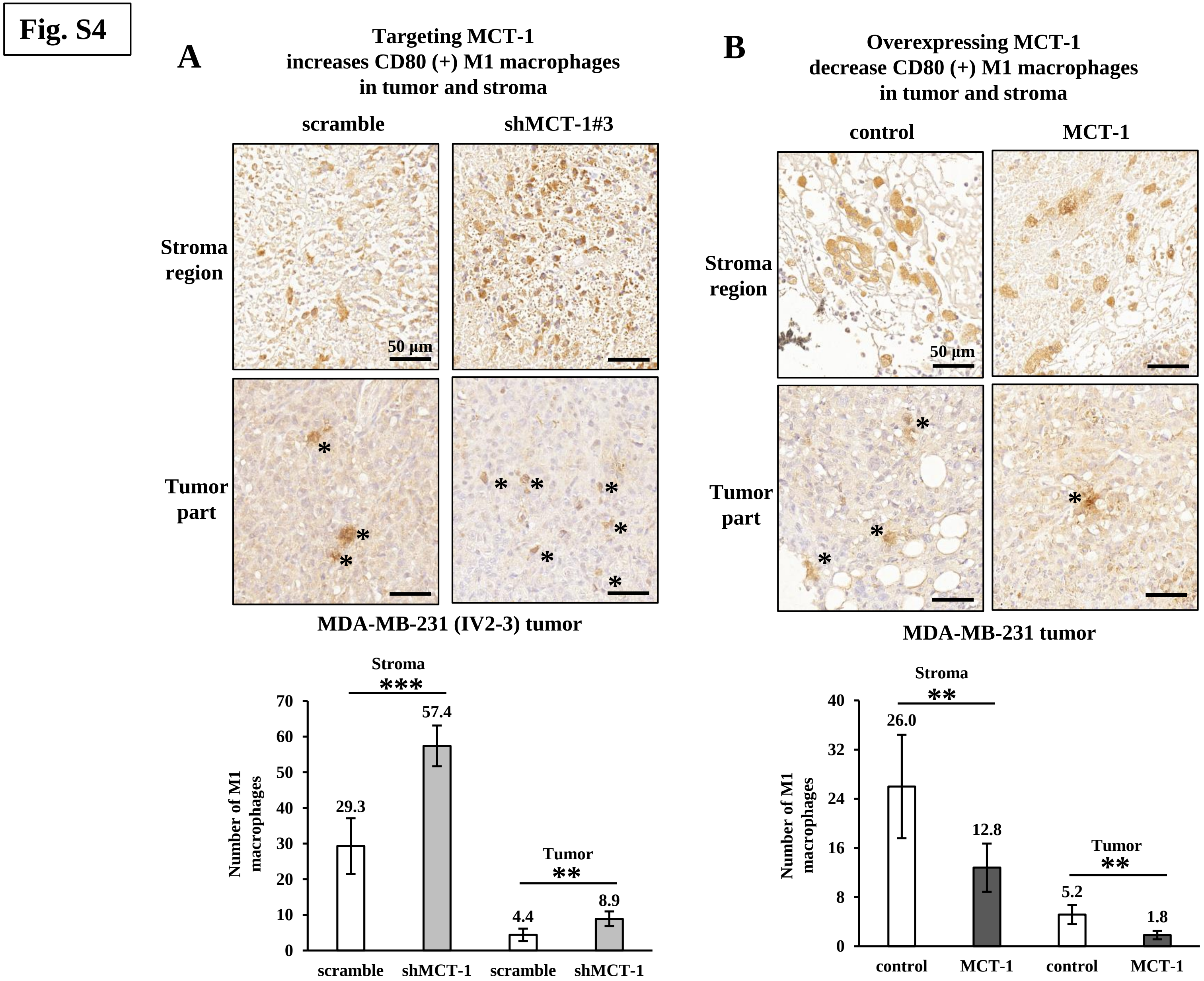

Supplementary Fig. S4. CD80-positive M1 macrophages enriched in the TME after MCT-1 knockdown. CD80-positive M1 macrophages accumulated in stroma and retained in MDA-MB-231 (IV2-3) tumors (scramble vs. shMCT-1#3) **(A)** and in MDA-MB-231 tumors (control vs. MCT-1) **(B)** were examined by immunohistochemistry studies. The images were captured with 40X objective lens and macrophage numbers were counted. (scramble, n=5 *vs.* shMCT-1#3, n=8; control, n=6 *vs.* MCT-1, n=6)). Scale bars, 50 µm
