# Supplementary material for: MCT-1/miR-34a/IL-6/IL-6R signaling axis promotes EMT progression, cancer stemness and M2 macrophage polarization in triple-negative breast cancer
Source: Mol Cancer. 2019 Mar 18;18:42. doi: 10.1186/s12943-019-0988-0 (PMC6421700; doi:10.1186/s12943-019-0988-0)
Supplement: Supplementary file 6 — Figure S6. Targeting MCT-1 and tocilizumab treatment together suppress 4 T1 cancer stemness. (PDF 144 kb) [file 12943_2019_988_MOESM6_ESM.pdf]

Fig. S6

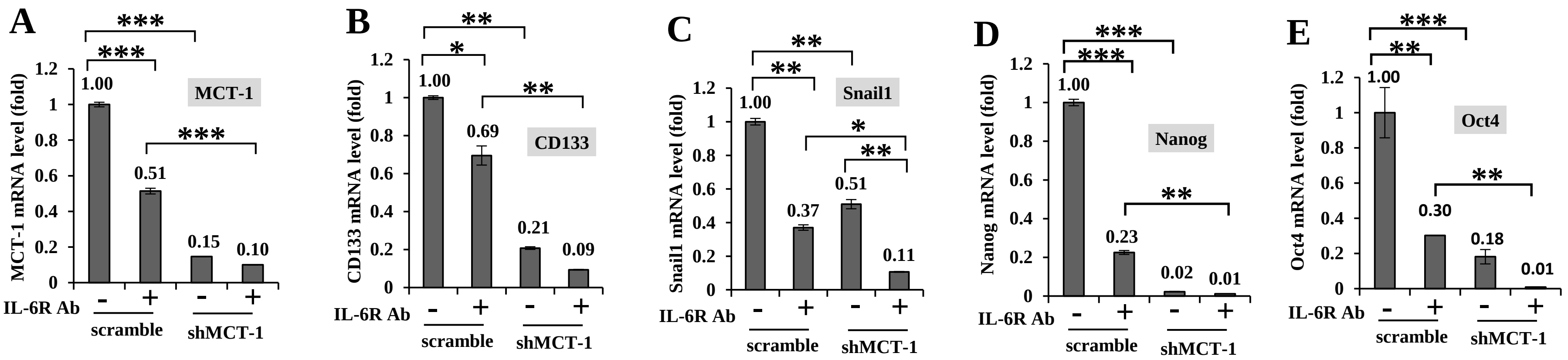

Supplementary Fig. S6. Targeting MCT-1 and tocilizumab treatment together suppress 4T1 cancer stemness. MCT-1 **(A)**, CD133 **(B)**, Snail **(C)**, Nanog **(D)** and Oct-4 **(E)** mRNA levels in the 4T1 mammospheres were evaluated after tocilizumab (200 µg/ml) treatment for 9 days. The results are expressed as the mean ± SD (n=3). (*p<0.05; **p<0.01; *** p<0.001).
