# Supplementary material for: MCT-1/miR-34a/IL-6/IL-6R signaling axis promotes EMT progression, cancer stemness and M2 macrophage polarization in triple-negative breast cancer
Source: Mol Cancer. 2019 Mar 18;18:42. doi: 10.1186/s12943-019-0988-0 (PMC6421700; doi:10.1186/s12943-019-0988-0)
Supplement: Supplementary file 7 — Figure S7. MicroRNA profiling in the MCT-1 pathway. (PDF 48 kb) [file 12943_2019_988_MOESM7_ESM.pdf]

Fig. S7

Silencing MCT-1 induces the tumor suppressor microRNAs

A

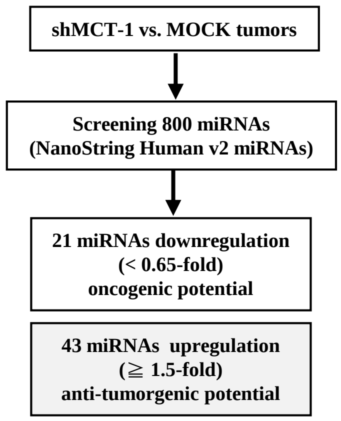

B

Decrease in shMCT-1 tumor

miRNA	fold
miR-1246	0.03
miR-223-3p	0.08
miR-1290	0.11
miR-494	0.14
miR-574-5p	0.14
miR-630	0.14
miR-3195	0.20
miR-320e	0.22
miR-451a	0.22
miR-521	0.31
miR-575	0.32
miR-522-3p	0.33
miR-4516	0.37
miR-517c-3p/ miR-519a-3p	0.40
miR-142-3p	0.42
miR-503	0.48
miR-340-5p	0.52
miR-601	0.56
miR-19a-3p	0.57
miR-4532	0.63
miR-19b-3p	0.65

Increase in shMCT-1 tumor

miRNA	fold
let-7f-5p	1.52
let-7b-5p	1.52
miR-429	1.52
let-7d-5p	1.54
miR-32-5p	1.54
miR-324-5p	1.58
miR-374a-5p	1.59
miR-26a-5p	1.59
miR-125a-5p	1.66
miR-30d-5p	1.66
miR-155-5p	1.67
miR-23b-3p	1.68
let-7e-5p	1.70
miR-99b-5p	1.70
miR-29b-3p	1.70
miR-29a-3p	1.71
miR-30a-5p	1.72
miR-152	1.77
miR-99a-5p	1.79
miR-1234	1.85
miR-196a-5p	1.86
miR-342-3p	1.87

miRNA	fold
miR-199a-5p	1.93
miR-497-5p	2.01
let-7c	2.02
miR-107	2.05
miR-203	2.12
miR-1260a	2.13
miR-30b-5p	2.23
miR-145-5p	2.24
miR-125b-5p	2.24
miR-411-5p	2.33
miR-34a-5p	2.34
miR-214-3p	2.38
miR-143-3p	2.38
miR-205-5p	2.55
miR-199a-3p/ miR-199b-3p	2.75
miR-720	2.79
miR-150-5p	2.94
miR-195-5p	2.96
miR-151a-3p	3.30
miR-4454	3.40

C

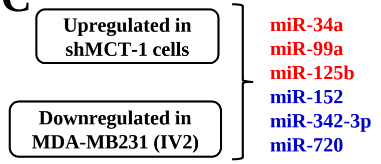

D

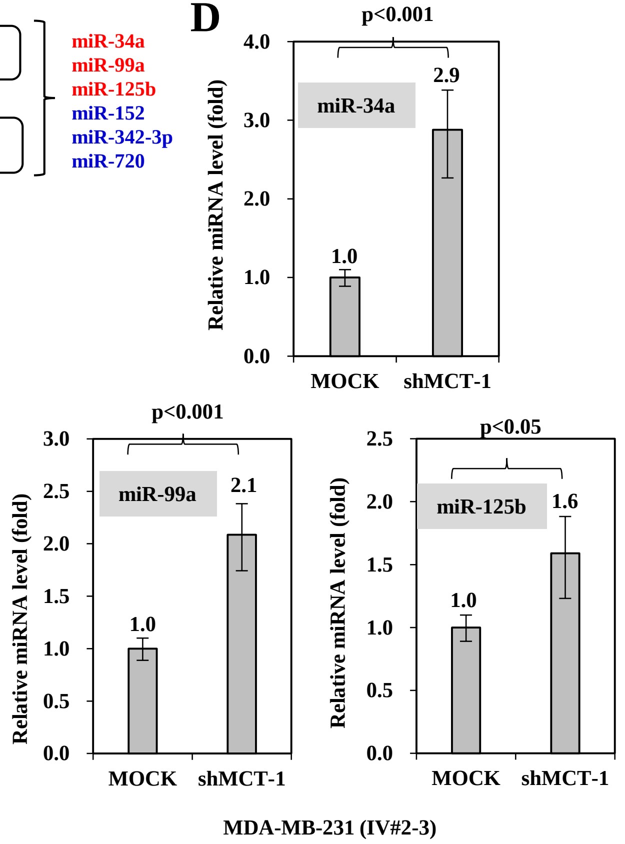

Supplementary Fig. S7. MicroRNA profiling in the MCT-1 pathway. **(A)** RNA samples of MDA-MB-468 tumors (scramble, shMCT-1) were used to screen the miRNA library. **(B)** Lists of the miRNAs that were specifically upregulated or downregulated in the shMCT-1 tumor. **(C)** The specific miRNAs upregulated in shMCT-1 cells were found to be downregulated in MDA-MB-231 (IV2-3). **(D)** Levels of miR-34a, miR-99b and miR-125b were confirmed to be induced after MCT-1 silencing compared with MOCK control by qRT-PCR. The results are expressed as the mean ± SD (n=3).
