# Supplementary material for: MCT-1/miR-34a/IL-6/IL-6R signaling axis promotes EMT progression, cancer stemness and M2 macrophage polarization in triple-negative breast cancer
Source: Mol Cancer. 2019 Mar 18;18:42. doi: 10.1186/s12943-019-0988-0 (PMC6421700; doi:10.1186/s12943-019-0988-0)
Supplement: Supplementary file 8 — Table S1. Primer sequences. (PDF 104 kb) [file 12943_2019_988_MOESM8_ESM.pdf]

Supplementary Table S1

Gene		Primer sequcence
β-actin	F	5′-CACCAGGGCGTGATGGTGGG-3′
	R	5′-GATGCCTCTCTTGCTCTGG GC-3′
ALDH1A1	F	5’-CAC GCC AGA CCT ACC TGT CC-3’
	R	5’-GCA GAG CTC CTC AGT TG-3’
CD24	F	5’-CTC ACA GAA CAA AGC AAG GGC-3’
	R	5’-GCC TAG CGC GAA CCC TCC-3’
CD44	F	5’-TTA CAG CCT CAG CAG AGC AC-3’
	R	5’-TGA CCT AAG ACG GAG GGA GG-3’
CD133	F	5’-GGG AAT GCC TAC TGG AA-3’
	R	5’-AAT TTG CAT GAA AGC ACA AGG-3’
EpCAM	F	5’-TGC TGG AAT TGT TGT GCT GG-3’
	R	5’-AGA TGT CTT CGT CCC ACG-3’
IL-6	F	5’- ATTCCTTCTTCTGGTCAGAAACC-3’
	R	5’-ACAAAGGATATTCAAACTGCATAGC-3’
IL-6R	F	5’- TTCACAGGGCACTGAGGAG -3’
	R	5’- GTCTTAATTTGGAGATGCAGGG -3’
MCT-1	F	5’-AGGCATTATCTTCATGCTGTCA-3’;
	R	5’-AATGATGGGCTGTGGCATAT-3’
Nanog	F	5’-TGG GAA GAA GCT AAA GAG CCA G-3’
	R	5’-GGA TGC TTC AAA GCA AGG CA-3’
Oct4	F	5’-TGA CCG CAT CTC CCC TCT AA-3’
	R	5’-TCT TCC CAG AGG GAG CTC AA-3’
Snail1	F	5’-GCG AGC TGC AGG ACT CTA AT-3’
	R	5’-GGA CAG AGT CCC AGA TGA GC-3’
SOX2	F	5’-CAT GAA GGA GCA CCC GGA TT-3’
	R	5’-TTA ATG TGC GCG TAA CTG TG-3’
F4/80	F	5’-CAATGAGTGCCTCACCAGCA-3’
	R	5’-TGGGCAAGCTCTTGGATCTG-3’
CD86	F	5’-GCTTTGCTTCTCTGCTGCTG-3’
	R	5’-GGCAGGTCTGCAGTCTCATT-3’
CD163	F	5’-CCGGGAGATGAATTCTTGCCT-3’
	R	5’-AGACACAGAAATTAGTTCAGCAGCA-3’
CD206	F	5’-CTGAATTGTACTGGTCTGTCCT-3’
	R	5’-GCTTAGATGTGGTGCTGTGG-3’
