# Supplementary material for: MCT-1/miR-34a/IL-6/IL-6R signaling axis promotes EMT progression, cancer stemness and M2 macrophage polarization in triple-negative breast cancer
Source: Mol Cancer. 2019 Mar 18;18:42. doi: 10.1186/s12943-019-0988-0 (PMC6421700; doi:10.1186/s12943-019-0988-0)
Supplement: Supplementary file 9 — Supplementary Methods. Cell culture. Plasmid construction and transfection. Targeting MCT-1 gene. Targeting IL-6 gene. Antibodies (Abs) and protein analysis. MCF-10A acinar morphogenesis. Cell invasion and migration assays. Gelatin zymography assay. Cell fractionation. Quantitative RT-PCR analysis of cancer stemness markers. ALDEFLUOR assay. Immunohistochemistry study. Quantification of miR-34a levels. Statistical analysis. (PDF 227 kb) [file 12943_2019_988_MOESM9_ESM.pdf]

Supplementary Materials and Methods

Cell culture

Non-tumorigenic human mammary MCF-10A cells were cultured in DMEM/F-12 medium (GIBCO, Grand Island, NY) supplemented with 5% horse serum (HS) (GIBCO), 20 ng/ml EGF (ProSpec-Tany TechnoGene Ltd., Rehovot, Israel), 10 μg/ml insulin (Sigma, St. Louis, MO), 0.5 μg/ml hydrocortisone (Calbiochem, Darmstadt, Germany), 100 ng/ml cholera toxin (Sigma), 100 units/ml penicillin (GIBCO) and 100 μg/ml streptomycin (GIBCO) in a 5% CO_2 incubator at 37°C. THP-1 monocytic leukemia cells, A549 lung cancer cells and TNBC cell lines (MDA-MB-231 and 4T1) were maintained in RPMI-1640 medium (Invitrogen, Carlsbad, CA) supplemented with 10% fetal bovine serum (FBS) (Life Technologies), 100 units/ml penicillin, 100 μg/ml streptomycin and 2 mM L-glutamine. Cells were maintained in an incubator with 5% CO_2 and 95% humidity at 37℃.

Plasmid construction and transfection

The full-length MCT-1 cDNA was cloned into the pLXSN retroviral vector as described [19]. The pLXSN vector (BD Biosciences, Clontech, Mountain View, CA) and pLXSN-MCT-1-V5 were transfected into PT67 packaging cells, using Lipofectamine 2000 (Invitrogen) as suggested by manufacture's instructions. Recombinant retroviral vector was packaged into infectious, replication-incompetent particles. Culture media containing the viral particles were collected for cellular transfection. The retrovirus containing supernatants were incubated with MCF-10A, MDA-MB231 and A549 cells for 24 hrs, replenished with fresh medium for another 24 hr, and then cultured under Neomycin G418 (400 μg/ml) selection for 2 weeks. Stable clones or mass cultures (control and MCT-1) were confirmed by V5-MCT-1 immunoblotting.

Targeting MCT-1 gene

The pGeneClip MCTS1 shRNA (#2: AGTCCGATGCCATGAACATAT, #3: TGCTGCAGTAGATACCATTGT for MDA-MB-231 (IV2-3) cells, TCCTTACAGTAAATGGAGAAT for 4T1 cells) (SA Biosciences Corp, Frederick, MD) or the scramble shRNA (ggaatctcattcgatgcatac) were transfected into MDA-MB-231 (IV2-3) or 4T1 cells using the jetPEI™ transfection reagent (Polyplus-transfection, New York, NY), followed by incubating with 0.5 μg/ml puromycin to select the stable lines with differential reduction of MCT-1.

To knockdown of MCT-1, SureSilencing pGeneClip MCT-1 shRNA and MOCK shRNA plasmids (SuperArray Biosciences Corporation) were stably transfected into MDA-MB-468 cells and cultured with 0.5 μg/ml puromycin.

Targeting IL-6 gene

The IL-6 shRNA (Santa Cruz, Dallas, TX) was a pool of 3 target-specific lentiviral vector plasmids each encoding 19-25 nucleotides shRNAs to knockdown IL-6 gene expression. The IL-6 shRNA and scramble vector (5 μg) were transfected into 293T cells and then the viral supernatants

were collected to transfected the indicated cells to establish the stable cell lines under 0.5 μg/ml puromycin selection for 2 weeks.

Antibodies (Abs) and protein analysis

Abs against EGFR (Tyr1068), EGFR, p-Src (Tyr416), Src, p-Stat3 (Ser727), p-Stat3 (Tyr705), Stat3, Slug, MMP2, MMP9, TIMP-2, TIMP-3, IL-6 and PD-L1 were purchased from Cell Signaling Technology, Inc. (Danvers, MA). Abs against ZEB1, ZEB2, Twist1 and TIMP-1 were purchased from Santa Cruz Biotechnology, Inc. (Dallas, Texas). Abs against V5-epitope (Invitrogen) and VEGF (Abcam, Cambridge, MA) as well as intrinsic MCT-1 (N1C3), β-actin, Snail, N-cadherin, vimentin, E-cadherin, SOX2, Nanog and EpCAM were all purchased from GeneTex (Irvine, CA). SDS-PAGE, Western blotting and the immunoprecipitation assays were conducted as described previously (15).

MCF-10A acinar morphogenesis

MCF-10A acini grown in Matrigel was studied as previously described (24). Briefly, growth factor-reduced Matrigels (BD Biosciences) were melted overnight at 4℃ and added evenly to an eight-well glass chamber slide (40 μl/well); then, the basement membrane was allowed to solidify for at least 15 min in a cell culture incubator. MCF-10A cells were harvested, washed in resuspension medium (DMEM/F12 medium with 20% horse serum) and gently resuspended in assay medium (DMEM/F12 with 2% horse serum, 0.5 μg/ml hydrocortisone, 100 ng/ml cholera toxin, and 10 μg/ml insulin and penicillin/streptomycin) without EGF at a single-cell suspension ($2.5x10^4$ cells/ml). The cell suspension (200 μl) and assay medium (200 μl) with 4% Matrigel and 10 ng/ml EGF were mixed and placed on the solidified Matrigel-coated chamber ($5x10^3$ cells/well). Cells were cultured in a 5% CO_2 humidified incubator at 37℃ and replenished with assay medium with 2% Matrigel and 5 ng/ml EGF every 4 days. After the cells formed clusters and then a hollow lumen at day 12, the medium was carefully removed, and the acini were immediately fixed with 2% paraformaldehyde in PBS for 20 min, permeabilized by 0.5% Triton X-100 in PBS for 10 min at 4℃, and rinsed thrice (15 min each) with PBS/glycine (130 mM NaCl, 7 mM Na_2HPO_4, 3.5 mM NaH_2PO_4, and 100 mM glycine). Samples were blocked by immunofluorescence (IF) buffer (PBS, 7.7 mM NaN_3, 0.1% bovine serum albumin, 0.2% Triton X-100, 0.05% Tween-20) plus 10% FBS for 1.5 h, incubated with the primary antibody (EGFR Ab) in DMEM/F12 medium with 10% FBS overnight at room temperature, gently rinsed thrice (20 min each) with IF buffer at room temperature and then incubated with an FITC-conjugated secondary antibody (Molecular Probes, Eugene, OR) at a 1:200 dilution in IF buffer/10% FCS for 1 h, followed by gently washing thrice (25 min each) with IF buffer. Nuclei were counterstained with 0.5 ng/ml 4′,6-diamidino-2-phenylindole (DAPI) (Sigma-Aldrich, St. Louis, Missouri) for 10 min and rinsed once with PBS for 15 min. Slides were mounted with Prolong Antifade Reagent (Molecular Probes) and analyzed under a confocal microscope.

Cell invasion and migration assays

Cancer cells (1×10^5) were resuspended in 500 µl of 1% FBS/RPMI and placed in the top chamber of a BioCoat Matrigel invasion chamber (Corning, Corning, NY) containing polycarbonate membrane with 8-µm pores, and 800 µl of 10% FBS/RPMI was placed in the bottom chamber. Cells were allowed to invade for 24 h at 37℃. The upper surface transwells were wiped off using a cotton swab to remove the uninvaded cells. The invaded cells on the reverse side of the membrane were fixed in ice-cold 75% methanol (Sigma-Aldrich) for 15 min, stained with 0.02% crystal violet for 30 min and counted under a light microscope at 200× magnification.

Cell invasiveness was also assayed by using a BD System Tumor Invasion Kit (BD Biosciences, San Jose, CA). Cells (5×10^4) were incubated in RPMI medium with 10 µg/ml of fluorescent dye DilC12(3) (BD Biosciences) for 2 h, rinsed with PBS, resuspended in 1% FBS/RPMI and allowed to invade the bottom chamber containing 10% FBS/RPMI for 24 h. The fluorescent invasive cells were measured directly using a FLUOstar Omega reader (BMG Labtech, Cary, NC) (excitation at 544 nm and emission at 590 nm).

Cancer cells (2×10^4) were incubated in 70 µl of 1% FBS/RPMI in a two-well Ibidi culture-insert (iBidi, Fitchburg, WI) for 24 h. After removing the insert, the cells were allowed to migrate for 24 h and were traced by a Leica AF6000 LX device equipped with a motorized stage in a heated, CO_2-regulated incubator. Images were taken every 10 min for 24 h and analyzed by MetaMorph 7.0 software.

Gelatin zymography assay

Cancer cells (1×10^6) were cultured in 1% FBS/RPMI medium for 24 h, and the CM was collected, mixed with the sample buffer and subjected to 8% SDS-PAGE containing 1 mg/ml gelatin. Afterward, the gels were washed twice with 2.5% Triton X-100/PBS for 30 min, incubated in developing buffer (50 mM Tris-HCl, pH 7.5, 200 mM NaCl, 5 mM CaCl2, 0.02% Brij 35) at 37℃ for 24 h, stained with Coomassie Brilliant Blue R-250 (Sigma-Aldrich) for 30 min and destained in a solution containing H_2O, methanol and acetic acid (50:40:10, v/v/v).

Cell fractionation

A Minute™ Plasma Membrane Protein Isolation and Cell Fractionation Kit (Invent Biotechnologies Inc., Plymouth, MN) was used to characterize cytosolic and membrane proteins. Cells (5×10^6) were incubated in 200 µl of buffer A on ice for 10 min, and the supernatants were transferred to the filter and centrifuged at 16,000 *g* for 30 sec. The filter was removed, and the supernatant was centrifuged at 700 *g* for 1 min. The supernatant was transferred to a 1.5-ml microcentrifuge tube and centrifuged at 16,000 *g* for 30 min at 4℃ to collect the cytosolic fraction. The pellet was dissolved in 100 µl PBS with protease inhibitor cocktail (Calbiochem-Merck, Darmstadt, Germany) to collect the membrane fraction. Heat shock protein 70 (Hsp70) and integrin-β1 (GeneTex, Irvine, CA) were the cytosolic and membrane fraction markers, respectively.

Quantitative RT-PCR analysis of cancer stemness markers

Mammosphere RNAs were extracted by the TRIzol^{TM} reagent (Invitrogen) and quantified by a Nano Drop ND1000 spectrophotometer (Thermo Fisher Scientific). RNA samples (1 μg) were reverse transcribed using a Maxima First Strand Synthesis Kit (Thermo Fisher Scientific). Reaction mixtures were performed at 25℃ for 10 min, 50℃ for 15 min and 85℃ for 5 min before chilling on ice for 10 min. Quantitative RT-PCR was conducted using SYBR Green Master Mix (Roche) and analyzed using an Applied Biosystems 7900HT Real-Time PCR system (Foster City, CA). The primers (Supplementary Table S1) of amplicons for human β-actin, ALDH1A1, CD24, CD44, CD133, EpCAM, IL-6, IL-6R, MCT-1, Nanog, Oct-4, Snai1 and Sox2 were synthesized (MDBio, Taipei, Taiwan) and designed according to the NCBI Probe database. The thermal reactions were initially denatured at 95℃ for 10 min, followed by 40 cycles of denaturation at 95℃ for 15 sec and annealing/extension at 60℃ for 1 min, and then were ended with a melting curve analysis. β-actin was a normalization control. Relative mRNA levels were calculated by the formula: ΔΔCT=ΔCt test group-ΔCt control group. The fold change in the gene was calculated using the formula $2^{-\Delta\Delta CT.}$

ALDEFLUOR assay

Aldehyde dehydrogenase activity was measured with an ALDEFLUOR kit (STEMCELL Technology, Vancouver, Canada). Mammosphere cells (5x10^5) were resuspended in 1 ml of the assay buffer with 5 μl ALDEFUOR reagent. One-half of the cells were incubated with diethylaminobenzaldehyde (DEAB) at a final concentration of 15 μM for 30 min at 37°C, and the other half were tested without DEAB. DEAB was an ALDH inhibitor that served as a control for reference fluorescence in each reaction. The ALDEFLUOR samples were analyzed by BD FACSCalibur flow cytometry and excited at 488 nm, and the emissions were determined by FL1 PMT (515-545 nm bandpass filter).

Immunohistochemistry study

Tumors were fixed in 10% formalin (Sigma-Aldrich), embedded in paraffin and sliced into 4-μm-thick sections before deparaffinization in xylene and hydration in ethyl alcohol (100%, 95%, 85%, and 75%) for 7 min each. Tumor sections were placed in citrate buffer (10 mM citric acid, 0.05% Tween 20, pH 6.0), heated for 20 min, rinsed twice with phosphate buffer saline (PBS) for 3 min and treated with 3% H_2O_2 in methanol for 15 min to block endogenous peroxidase activity. After blocking with the background blocker (Enzo Biochem, Inc., Farmingdale, NY), Abs against MCT-1 (GTX117793) (1:200) (GeneTex, Irvine, CA), E-cadherin (#610182) (1:100) (BD Biosciences), vimentin (#5741) (1:100) (Cell Signaling Technology, Inc.), CD31 (ab28364) (1:50) (Abcam), F4/80 (GTX26640) (1:50) (GeneTex), CD80 (Abcam) (ab64116) (1:200) and CD163 (ab182422) (1:100) (Abcam) were diluted in PBS as indicated. Samples were stained with the primary Abs for 2 h, incubated with the secondary Ab (goat anti-rabbit IgG, H+L, #31460) (Thermo Fisher Scientific, Waltham, MA) for 1 h, reacted with a liquid DAB+ Substrate Chromogen System (Agilent Dako, Santa Clara, CA) and counterstained

with Gill No. 2 hematoxylin (Thermo Fisher Scientific). Slides were dehydrated, mounted with Histokitt mounting medium (Glaswarenfabrik Karl Hecht GmbH & CO., Germany) and observed under a Nikon Optiphot-2 Upright Microscope (Nikon Corporation, Tokyo, Japan) using 40× objective lens. Images were analyzed by an automatic digital slide scanner system Pannoramic MIDI II (3DHISTECH, Ltd., Budapest, Hungary). The relative staining strength of the MCT-1 protein was defined as absent (-), minor (+), distinct (++) and strong (+++). Low and high MCT-1 amounts were defined as absent/minor (-/+) and distinct/strong (++/+++), respectively.

Quantification of miR-34a levels

Total cellular RNA was isolated by TRIzol reagent (Invitrogen), and the reverse transcription reactions for miR-34a were performed using the Maxima First Strand cDNA Synthesis Kit for qRT-PCR (Thermo Fisher Scientific) with the gene specific primer (5'-GTTGGCTCTGGTGCAGGGTCCGAGGTATTCGCACCAGAGCCAACACAACC-3'). The miR-34a levels were evaluated by qRT-PCR conducted using the primers (forward: 5'-CGGTGGCAGTGTCTTAGCT-3'; reverse: 5'-GTGCAGGGTCCGAGGT-3') and FastStart Universal SYBR Green Master (Rox) (Roche, Mannheim, Germany). Thermal cycling conditions were performed as described above. Small nucleolar RNA (RNU6B) levels detected by qRT-PCR using the primers (forward: 5'-CTCGCTTCGGCAGCACA-3'; reverse: 5'-AACGCTTCACGAATTTGCGT-3') acted as an internal control. Relative miR-34a levels were calculated by the formula: ΔΔCT=ΔCt test group-ΔCt control group. The miR-34a levels were calculated using $2^{-\Delta\Delta Ct}$.

Statistical analysis

The Kaplan–Meier method was used to estimate the survival curve and compare the survival rates of two groups using the log rank test. The Fisher exact probability independent test was employed to analyze the impact in the clinical immunohistochemistry studies. The Chi-square test analyzed the correlation between MCT-1 with IL-6 and with IL-6R gene expression in the breast cancer cDNA arrays. Pearson's correlation coefficient identified the statistical relevance between MCT-1 with IL-6 and IL-6R gene expression in the Oncomine database. One-way ANOVA with a post hoc two-tailed t-test was used to calculate the statistical significance of pairwise comparisons of cancer cell invasion/migration, gene expression and cancer stemness.
